# A Symphony of Signals: Intercellular and Intracellular Signaling Mechanisms Underlying Circadian Timekeeping in Mice and Flies

**DOI:** 10.3390/ijms20092363

**Published:** 2019-05-13

**Authors:** Sara Hegazi, Christopher Lowden, Julian Rios Garcia, Arthur H. Cheng, Karl Obrietan, Joel D. Levine, Hai-Ying Mary Cheng

**Affiliations:** 1 Department of Biology, University of Toronto Mississauga, Mississauga, ON L5L 1C6, Canada; sara.hegazi@mail.utoronto.ca (S.H.); chris.lowden@mail.utoronto.ca (C.L.); julian.riosgarcia@mail.utoronto.ca (J.R.G.); ahh.cheng@mail.utoronto.ca (A.H.C.); joel.levine@utoronto.ca (J.D.L.); 2 Department of Cell and Systems Biology, University of Toronto, Toronto, ON M5S 3G5, Canada; 3 Department of Neuroscience, Ohio State University, Columbus, OH 43210, USA; obrietan.1@osu.edu

**Keywords:** circadian rhythms, central pacemaker, suprachiasmatic nucleus, *Drosophila*, neurotransmitters, neuropeptides, entrainment, synchrony, intercellular and intracellular signaling, protein kinases

## Abstract

The central pacemakers of circadian timekeeping systems are highly robust yet adaptable, providing the temporal coordination of rhythms in behavior and physiological processes in accordance with the demands imposed by environmental cycles. These features of the central pacemaker are achieved by a multi-oscillator network in which individual cellular oscillators are tightly coupled to the environmental day-night cycle, and to one another via intercellular coupling. In this review, we will summarize the roles of various neurotransmitters and neuropeptides in the regulation of circadian entrainment and synchrony within the mammalian and *Drosophila* central pacemakers. We will also describe the diverse functions of protein kinases in the relay of input signals to the core oscillator or the direct regulation of the molecular clock machinery.

## 1. Introduction

Circadian (from the Latin phrase “circa diem,” meaning “about a day”) clocks are biological timekeeping mechanisms that have evolved to enable organisms to coordinate behavior and physiological processes to the cyclical changes occurring within their environment [1].

**Figure 1:**
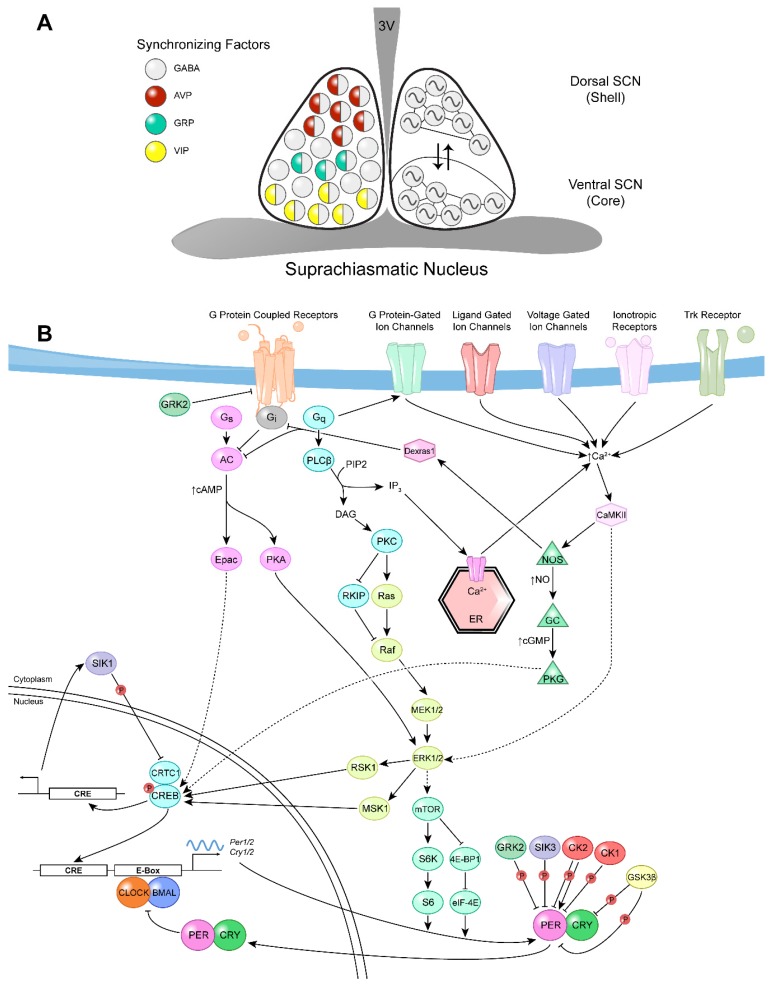
The mammalian suprachiasmatic nucleus: neurochemical composition and canonical signaling pathways that regulate the core oscillator and photic entrainment. (**A**) Neurochemical composition of the SCN. The SCN is divided into ventral (core) and dorsal (shell) regions. Most, if not all, of the ~20,000 SCN cells are GABAergic but differ in their neuropeptide content. VIP and AVP delineate the core and shell regions, respectively. GRP-expressing neurons are primarily localized in the medial core. Prokineticin 2 is another SCN neuropeptide that is highly expressed by core and shell SCN neurons. (**B**) Canonical signaling pathways within the SCN. The figure depicts the major signaling pathways and protein kinases that have so far been shown to function within the SCN. Various neuropeptides and neurotransmitters impinging on SCN neurons can activate receptors and ion channels on the plasma membrane to trigger intracellular signaling events. Activation of G-protein coupled receptors (GPCRs) can signal via G_s_, G_i_, and G_q_ proteins to activate adenylyl cyclase (AC), inhibit AC, or activate phospholipase Cβ (PLCβ), respectively. AC stimulates the production of cAMP, which in turn activates protein kinase A (PKA) and exchange protein activated by cAMP (Epac). CREB-mediated transcription can be induced by PKA and Epac. PLCβ catalyzes the hydrolysis of phosphatidylinositol 4,5-bisphosphate into diacylglycerol (DAG) and inositol 1,4,5-trisphosphate (IP_3_). DAG activates protein kinase C at the plasma membrane, whereas IP_3_ diffuses into the cytosol to induce the release of intracellular Ca^2+^ stores from the endoplasmic reticulum (ER). The rise in cytosolic Ca^2+^ levels can also be induced by ionotropic receptors, voltage-gated ion channels, G-protein gated ion channels, and receptor tyrosine kinases. Downregulation of GPCR signaling is achieved by phosphorylation of the receptor by G protein-coupled receptor kinase 2 (GRK2). Receptor-mediated Ras activation at the plasma membrane stimulates the mitogen-activated protein kinase pathway (RAF, MEK1/2, ERK1/2). PKC facilitates Ras/MAPK signaling, either by activating Ras or derepressing RKIP-mediated inhibition of Raf via phosphorylation of RKIP. MAPK/ERK activates p90 ribosomal S6 kinase 1 (RSK1) and mitogen- and stress-activated protein kinase 1 (MSK1), which in turn stimulate CREB-mediated transcription. MAPK/ERK is also an upstream activator of mammalian target of rapamycin (mTOR), which promotes translation by activating p70 S6 kinase (p70S6K) and inhibiting eukaryotic translation initiation factor 4E (eIF4E)-binding protein 1 (4E-BP1)-mediated repression of eIF4E. In terms of Ca^2+^ signaling, Ca^2+^-induced activation of Ca^2+^/calmodulin-dependent protein kinase II (CaMKII) can couple to cyclic guanosine monophosphate (cGMP) production through the nitric oxide synthase (NOS)/guanylyl cyclase (GC) pathway. cGMP activates protein kinase G (PKG), which promotes CREB-mediated transcription. The NOS pathway also activates the small G protein, Dexras1, which inhibits GPCR-mediated G_i_ activation and indirectly inhibits AC through ligand-independent activation of G_i_. Many upstream signaling events converge on the MAPK/ERK pathway, a pivotal player in photic entrainment via its effects on CREB. Salt-inducible kinase 1 (SIK1) is a CRE-inducible gene that acts as a feedback inhibitor of CREB signaling through suppression of CREB-dependent transcription coactivator 1 (CRTC1). Finally, a number of protein kinases have been shown to phosphorylate clock proteins, PERIOD and CRY: these include casein kinase 1 and 2 (CK1 and CK2, respectively), glycogen synthase kinase 3 (GSK3), SIK3, and GRK2. Dashed lines denote indirect interactions.

In mammals, the circadian system is partitioned into a hierarchy of peripheral oscillators that are governed by a central pacemaker known as the suprachiasmatic nucleus (SCN) [2,3]. Located within the anterior hypothalamus, the SCN is a bilateral structure comprised of ~20,000 GABAergic (GABA; γ-aminobutyric acid) neurons (Figure 1A) [4,5]. It is anatomically subdivided into the dorsomedial “shell” and ventrolateral “core” regions, where each region is characterized by a distinct functional role and expression pattern of neuropeptides [5,6]. The ventral SCN primarily expresses gastrin-releasing peptide (GRP) and vasoactive intestinal polypeptide (VIP) and is directly responsive to photic input [7,8,9,10]. In contrast, the dorsal SCN expresses arginine vasopressin (AVP) and is crucial in maintaining rhythmicity [6]. Extensive intercellular communication within and between SCN compartments occurs via direct reciprocal innervations that enable the numerous oscillating clock cells to sustain a single, uniform output.

A core clock mechanism of transcription-translation feedback loops (TTFLs) drives rhythms in each of the clock neurons of the SCN. Within the TTFL, the transcription factors, circadian locomotor output cycles kaput (CLOCK) and brain and muscle aryl hydrocarbon receptor nuclear translocator-like protein 1 (BMAL1), bind E-box elements within the promoter regions of the Period (*Per*) and Cryptochrome (*Cry*) clock genes to drive their expression. In the cytoplasm, several post-transcriptional, translational and post-translational mechanisms act to regulate rhythmic protein expression in accordance with the day/night cycle. The loop eventually closes when the PER and CRY proteins translocate to the nucleus and inhibit the transcriptional activity of the CLOCK-BMAL1 complex (Reviewed in [11]). 

In *Drosophila*, the circadian system is made up of a central oscillator network in the brain, and several peripheral oscillators. Daily rhythms in behavior are dictated by the central pacemaker circuit, which has recently been modeled as a network of multiple, independent oscillators characterized by a high degree of anatomical, functional and neurochemical diversity [12,13]. Under this model, individual oscillators or clusters of oscillators exert autonomous control over different aspects of activity rhythms, such that no one cluster on its own is sufficient for the coherent function of the network as a whole [12,13,14]. Clock neurons in the *Drosophila* brain, like those in the SCN, employ various neurotransmitters and neuropeptides to signal temporal information within and between cell clusters. Extensive intercellular communication within and between clusters is required for the generation of coherent behavioral rhythms. 

**Figure 2:**
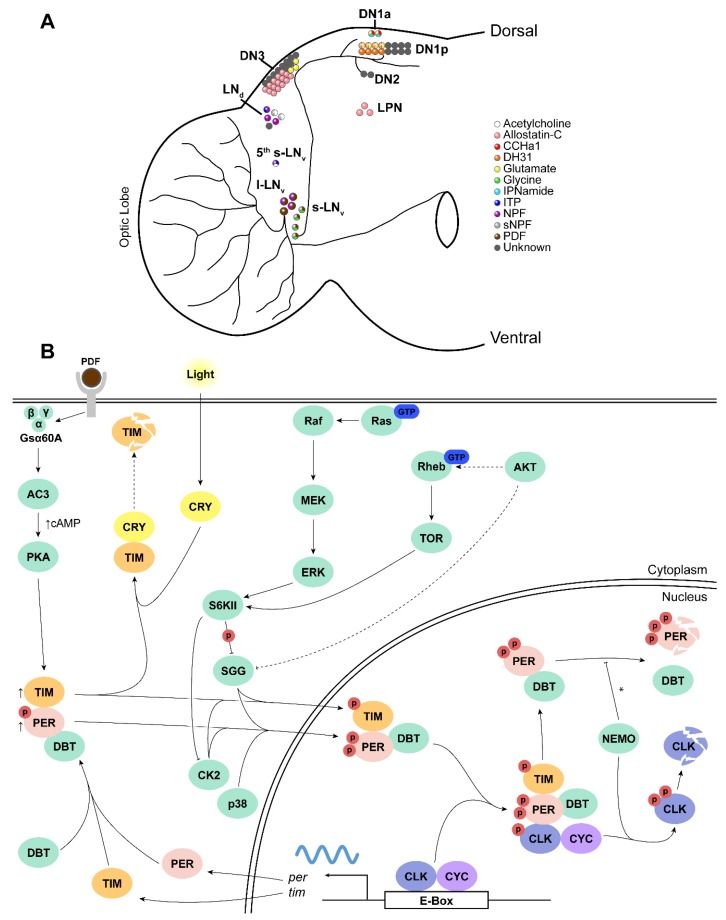
The central clock network in *Drosophila*: neuronal clusters, neurochemical composition, and clock regulation by protein kinases. (**A**) Classification and neurochemical composition within the adult *Drosophila* central pacemaker. The clock network is composed of ~150 neurons that include the small and large ventral lateral neurons (s-LN_v_s and l-LN_v_s, respectively), dorsal lateral neurons (LN_d_s), dorsal neurons (DNs), and lateral posterior neurons (LPNs). Different neurochemicals colocalize in the same neuron within a cluster. The PDF-expressing s-LN_v_s project into the dorsal protocerebrum, and the l-LN_v_s project contralaterally and into the optic lobe. Glutamate, DH31 and Allostatin-C are expressed in overlapping and non-overlapping subsets of DN_1p_ neurons. While Allostatin-C and glutamate have been shown to co-localize within the same neurons, it is not clear whether DH31 co-localizes with either Allostatin-C or glutamate in the same DN_1p_ neurons. CCHa1, CCHamide1; DH31, Diuretic Hormone 31; ITP, Ion Transport Peptide; NPF, Neuropeptide F; sNPF, Short Neuropeptide F; PDF, Pigment Dispersing Factor. (**B**) Regulation of the clockwork via protein kinases in the *Drosophila* pacemaker. The primary clock feedback loop where CLK/CYC dimers initiate the transcription of *per* and *tim* genes. The phosphorylated PER/TIM complex then translocates to the nucleus to repress CLK/CYC activity. Several protein kinases are involved in mediating the nuclear translocation and degradation of PER and TIM within the nuclear and cytoplasmic compartments. In the nucleus, kinases act to repress the CLK/CYC transcriptional complex via the phosphorylation and degradation of CLK. PER and TIM must also undergo degradation in the nucleus to reset the loop. The asterisk (*) refers to the action of NEMO in priming PER for DBT-mediated phosphorylation. Also shown is the role of PDF-PDFR signaling in stabilizing PER and TIM proteins. See text for detailed description of the depicted pathways. AC3, Adenylyl Cyclase 3; AKT, Protein Kinase B; cAMP, cyclic adenosine monophosphate; CK2, Casein Kinase 2; CLK, Clock; CRY, Cryptochrome; CYC, Cycle; DBT, Doubletime; Gsα60A, stimulatory G protein α subunit 60A; GTP, guanosine triphosphate; MAPK, Mitogen-Activated Protein Kinase; MEK, MAPK/ERK Kinase; NEMO, NEMO kinase; p38, p38 MAPK; PER, Period; PKA, Protein Kinase A; Ras, Ras-GTPase; Rheb, Rheb GTPase; SGG, Shaggy; S6KII, Ribosomal S6 Kinase II; TIM, Timeless; TOR, Target of Rapamycin. Phosphate groups are depicted in red circles (P); dashed lines show indirect effects through other signaling molecules; dissociated proteins indicate degradation; upward pointing arrows placed beside molecules show stabilization and/or accumulation.

The *Drosophila* central pacemaker is made up of ~150 clock neurons in the brain (Figure 2A). These are divided into several clusters based on neuroanatomy: the large and small ventral lateral neurons (pigment dispersing factor (*Pdf*)-positive l-LN_v_ and s-LN_v_, respectively, and the *Pdf*-negative 5^th^ s-LN_v_), the dorsal lateral neurons (LN_d_), the lateral posterior neurons (LPN), and 3 classes of dorsal neurons (DN_1_, DN_2_, DN_3_) [15]. Owing to the conserved nature of circadian clocks, each clock neuron operates with a similar TTFL mechanism whereby the CYCLE (CYC) and CLOCK (CLK) transcription factors drive expression of the period (*per*) and timeless (*tim*) genes. PER/TIM dimers then translocate to the nucleus to repress their own transcription by inhibiting the CLK/CYC dimer (Reviewed in [11]).

As crepuscular organisms, flies maintained under an environmental light-dark (LD) cycle display a bimodal activity profile characterized by morning (M) and evening (E) components that reflect anticipatory behavior preceding lights-on and lights-off, respectively [16,17]. The morning component is primarily driven by the s-LN_v_ neurons (morning cells, or M cells), whereas the evening component is controlled by the PDF-negative 5^th^ LN_v_ and the LN_d_s (Evening cells, or E cells) [18,19,20]. A subset of DN_1_s and l-LN_v_s have also been implicated in LD activity (e.g., [21,22,23,24,25,26]). However, recent findings demonstrate that this classification of M and E oscillators is context-dependent and not as strict as previously thought [14,20,27]. In addition, the M cells are recognized for their pivotal role in the generation of free-running rhythms via the synchronizing actions of PDF [18,28,29,30,31,32]. The l-LN_v_s have been extensively studied for their role in light-mediated arousal and wakefulness [21,33,34,35]. The dorsal neurons modulate activity rhythms via interactions with other clock neurons: they have been demonstrated to modulate LD activity, free-running rhythms (under constant dark [DD] and constant light [LL] conditions), and temperature preference rhythms (e.g., [19,26,36,37,38,39,40]). The role of the LPN cluster is poorly characterized, although recent studies have suggested a function in promoting sleep and modulating evening activity [41,42]. For the precise temporal modulation of activity rhythms, both M and E cells act in coordination with one another and with other clock neurons, communicating via neurotransmitters and neuropeptides. 

The central clocks of mammals and flies can be entrained by photic and non-photic zeitgebers (from the German terms “zeit” [meaning “time”] and “geber” [meaning “giver”]), with light being the most potent entraining cue. In mammals, the retinohypothalamic tract (RHT) of the optic nerves is responsible for monosynaptically relaying non-visual, photic information from the retina to the retinorecipient core SCN region [8,43]. Retinal ganglion cells of the RHT co-store the excitatory neurotransmitter, glutamate, and the neuropeptide, pituitary adenylate cyclase-activating peptide (PACAP), both of which play vital roles in photic signaling to the SCN [44,45,46,47,48,49]. The core SCN subsequently communicates phase-resetting signals to the shell region. In this way, the core SCN governs clock entrainment and the majority of light-induced molecular and behavioral circadian responses. In contrast to the core region, which displays low amplitude oscillations in clock gene expression, the shell SCN is characterized by high amplitude circadian gene expression and is indispensable for maintaining free-running rhythms at the behavioral and molecular levels [5,6,50]. 

In *Drosophila*, circadian photoreception occurs via three independent pathways that include: (1) the compound eyes and ocelli, (2) the extraretinal Hofbauer-Buchner (H-B) eyelets, and (3) the blue-light photoreceptor CRYPTOCHROME (CRY) (Reviewed in [51]). Light transduction by the first two photoreceptive organs is achieved through synaptic connections with clock neurons. In the context of visual photoentrainment, clock neurons receive light input from the compound eyes through histamine-dependent and histamine-independent mechanisms via photoreceptors or interneurons, and from the H-B eyelet photoreceptors through cholinergic and histaminergic transmission [25,51,52,53,54]. Although circadian photoreception in *Drosophila* exhibits some features of hierarchical organization as evidenced by the role of LN_v_s in the circuit-wide relay of photic input, in contrast to the mammalian system, it is capable of independently occurring in each cluster of clock neurons either through the aforementioned visual structures or cell-autonomously via CRY [25,51,52,55]. In flies, CRY is widely expressed in various clock neurons [56,57]. However, unlike the mammalian clock, it is not part of the core clockwork and instead serves as a photoreceptor for circadian entrainment [58]. Light penetrating the fly cuticle can directly entrain CRY-expressing neurons via a mechanism that involves the degradation of TIM [59]. 

The contribution of the central pacemakers to peripheral oscillations differs between mammals and flies but shares some common features. Under normal conditions, the SCN serves as the master orchestrator of coherent, rhythmic activity throughout the body. It primarily acts to synchronize “slave” oscillators in the periphery to environmental cycles via neural and humoral output pathways. Synchronized peripheral clocks then control local rhythms in physiology and behavior. Ultimately, in the absence of input from the SCN, constituent cellular oscillators of peripheral tissues rapidly desynchronize due to weak coupling, leading to damped oscillations at the tissue level [1,60,61]. This demonstrates the functional hierarchy within the multi-oscillator system of the mammalian clock. In some instances, however, peripheral clocks can maintain a rhythm that is decoupled from and oscillating independently of the SCN, as is the case for the liver, kidney, heart and pancreas clocks, under temporally restricted feeding schedules [62,63]. Similarly, chronic administration of the psychostimulant, methamphetamine, is capable of driving overt rhythms in physiology and behavior independently of the SCN [64]. 

In *Drosophila*, the cell-autonomous, entraining effects of CRY extend to many peripheral oscillators that drive rhythms in feeding, olfaction and eclosion, among other processes [65,66]. Thus, unlike the mammalian circadian system, synchrony within the fly circadian network is largely maintained by cell-autonomous responses to common entraining cues, such that peripheral rhythmic activity is minimally dependent on central input, but rather occurs in a tissue-specific manner [66,67]. For instance, the fat body clock, similar to the mammalian liver, can independently maintain molecular oscillations under restricted feeding schedules [68]. Similarly, olfaction rhythms mediated by antennal neurons and molecular oscillations in the Malpighian tubules persist in the absence of central clock input [67,69]. However, a few clocks are either modulated or, in exceptional cases, driven by the central clock: an example of each are the oenocytes and the prothoracic gland, respectively [70,71]. 

Overall, circadian systems in mammals and flies are largely regulated by temporal coordination within the central pacemaker network of functionally distinct autonomous oscillators, and between central and peripheral clocks. Nevertheless, the mammalian circadian system demonstrates a relatively higher degree of hierarchy within the extensively-coupled SCN core and shell compartments, and through master control of the SCN over peripheral clocks. On the other hand, in *Drosophila*, the central pacemaker network of flexibly coupled clock neurons has relatively less control over peripheral rhythms, reflective of a less hierarchical arrangement. 

Given that the central pacemaker is indispensable for the optimal functioning of behavioral and physiological processes, its proper regulation is of paramount importance. TTFL mechanisms provide the basis for the rhythmic nature of circadian pacemakers of both flies and mice. However, in order for the central clock to generate a coherent output that conveys accurate time-of-day information, there must be mechanisms that couple constituent cellular oscillators within the central clock network to one another as well as mechanisms that couple the clock to the environment. The combined actions of various intercellular and intracellular pathways serve to maintain precise synchrony with geophysical time, while also conferring on the central pacemaker an intrinsic resistance to stochastic environmental and genetic perturbations. In this review, we will examine the inter- and intra-cellular signaling mechanisms that regulate central pacemakers in mice and flies, and their roles in maintaining accurate and coherent circadian timekeeping. The review places special emphasis on the role of neurochemical messengers and protein kinases in entrainment and circadian pacemaking.

## 2. Neuromodulation of Entrainment of the Mammalian Central Pacemaker

### 2.1. Transmission of Photic Information to the SCN: The Role of Neurotransmitters and Neuropeptides

#### 2.1.1. Glutamate

The role of glutamate in photic signaling to the SCN is well established. All three glutamate receptor subtypes, namely α-amino-3-hydroxy-5-methyl-4-isoxazolepropionic acid (AMPA), kainate, and N-methyl-D-aspartate (NMDA), are expressed by SCN neurons [72,73,74]. The addition of glutamate to SCN tissue explants has been shown to induce neuronal firing [75], and optic nerve stimulation results in glutamate release [76]. Pharmacological experiments have demonstrated that these effects are largely dependent on NMDA and non-NMDA ionotropic glutamate receptors, since application of selective receptor antagonists in vitro and in vivo reversibly attenuated SCN neuronal activity that was induced by RHT stimulation [77]. In addition, treatment with NMDAR antagonists perturbed light-induced nocturnal phase-shifts of wheel-running activity as well as SCN neuronal activity [78,79]. Treatment with NMDA or AMPA agonists during the subjective night recapitulated the phase-shifting effects of light in vitro [78].

In addition to ionotropic glutamatergic signaling, metabotropic glutamate receptors (mGluRs) have been shown to modulate SCN neuronal activity. All eight subtypes of mGluRs are expressed within the SCN [80]. mGluR activation increased the firing rate of clock neurons in vitro, particularly those neurons found in the ventrolateral SCN compartment [81]. In autaptic SCN cultures, activation of mGluRs attenuated the presynaptic release of GABA and consequent GABA-dependent modulation of neuronal activity [82]. These results indicate that excitatory firing via mGluR signaling is mediated through modulation of responsiveness to GABA [82].

A recent study added yet another layer of complexity to the current view of glutamatergic modulation of the central clock. Brancaccio et al. (2017) demonstrated that SCN astrocytes constituted the other “half” of the circadian clock by releasing glutamate and elevating extracellular glutamate levels during the subjective night [83]. Increased levels of extracellular glutamate would activate pre-synaptic NMDAR2C complexes on dorsomedial SCN neurons, thereby increasing the inhibitory tone within the circuit and suppressing SCN neuronal activity [83]. During the subjective day, reduced release of glutamate by astrocytes, and the consequent reduction in extracellular glutamate levels, would lead to de-repression of the spontaneous activity of SCN neurons [83].

Several intracellular signaling pathways have been implicated in the downstream effects of glutamatergic signaling to the SCN. Glutamate release from the RHT activates both NMDA and non-NMDA receptors in neurons within the ventrolateral SCN, increasing intracellular calcium (Ca^2+^) levels through phase-dependent Ca^2+^ channel activation [84]. The phase-dependence of Ca^2+^ signaling may comprise a part of the gating mechanism that restricts the phase-shifting effects of light or glutamate to the nocturnal domain [84]. Depending on the nocturnal phase, different signaling cascades may mediate molecular clock resetting. Glutamate stimulation in the early night induces Ca^2+^ influx through T-type voltage gated Ca^2+^ channels (VGCCs) and ryanodine receptor-gated intracellular Ca^2+^ stores [85,86]. In contrast, L-type Ca^2+^ channels are preferentially activated during the late night [85]. In addition to calcium, other signaling events that are induced by photic stimulation also exhibit phase dependence. For example, the phase-delaying effects of early-night glutamate treatment involve the activation of cyclic adenosine monophosphate (cAMP)/protein kinase A (PKA) signaling, resulting in increased *Per1* expression [87]. In contrast, the phase-advancing effects of late-night glutamate stimulation are mediated by cyclic guanosine monophosphate (cGMP)/protein kinase G (PKG) signaling and are attenuated by cAMP/PKA activation [87,88]. The generation of nitric oxide (NO) also appears to be integral to glutamate-induced phase-resetting, given that intracerebroventricular (ICV) injections of a NO synthase inhibitor attenuated the phase-shifting effects of light [89,90]. Lastly, brain derived neurotrophic factor (BDNF), acting via the tyrosine kinase B (TrkB) receptor, has been shown to augment glutamate-induced phase shifts of neuronal activity in the SCN [91,92].

#### 2.1.2. Pituitary Adenylate Cyclase-Activating Polypeptide (PACAP)

PACAP serves as a critical modulator of glutamatergic signaling within the SCN, where it functions to fine-tune the magnitude of the photic response in a time-of-day-dependent manner. Application of PACAP to SCN tissue explants augmented the magnitude of glutamate-induced phase delays of neuronal firing rhythms during the early night, but inhibited glutamate-induced phase advances during the late night [47]. Consistent with the latter observation, ICV infusions of a PACAP antibody enhanced the light-evoked phase advance in wheel-running activity [47]. The effects of PACAP on glutamate-induced phase shifts likely stem, in part, from changes in intracellular Ca^2+^ levels. In vitro application of PACAP can augment the glutamate-induced increase in intracellular Ca^2+^ via cAMP-independent mechanisms that include opening of voltage-activated L-type Ca^2+^ channels as well as AMPA/kainate signaling [93,94]. PACAP-mediated increases in Ca^2+^ transients were shown to require protein kinase C (PKC) and p42/p44 mitogen-activated protein kinase (MAPK) signaling [94,95]. On the other hand, PACAP diminishes the rise in intracellular Ca^2+^ that is elicited by glutamate-evoked mGluR activation in a manner that is dependent on cAMP signaling [95]. 

The phase-shifting effects of PACAP alone, in the absence of glutamate co-administration, have been widely documented. Daytime application of PACAP-38, the dominant, biologically active form of PACAP [96], advances the phase of SCN firing rhythms in vitro [97]. This response seems to be mediated by the induction of cAMP-PKA signaling downstream of PACAP receptor type 1 (PACAP-R1/PAC1) activation in ventrolateral SCN neurons [46,97]. Notably, this effect is specific to PACAP, since VIP fails to elicit a similar response [97]. The application of PACAP-38 during the early night delays the phase of SCN firing rhythms, recapitulating the effects of early-night light exposure and supporting a prominent role of PACAP in photic entrainment [46]. These phase-delaying effects of PACAP are likely mediated by a potentiation of glutamatergic currents involving NMDA receptors [46]. 

PACAP acts through one of two receptors, the PACAP type 1 receptor (PAC1), which is highly selective for PACAP [98], and the VPAC2 receptor, which is equally sensitive to PACAP and VIP [99]. However, existing evidence suggests that, within the SCN, PACAP predominantly signals through the PAC1 receptor [93,97,100]. *PAC1^−/−^* mice exhibited a potentiation in early-night light-induced phase delays of locomotor activity rhythms but, surprisingly, an attenuation in the photic induction of the clock genes *Per1* and *Per2*, as well as the immediate-early gene (IEG), *c-Fos* [101]. These results indicate that disrupting PAC1 signaling triggers a dissociation between light-induced behavioral phase shifts and clock gene or IEG induction [101]. In *PAC1^−/−^* mice, a late-night light pulse resulted in normal induction of *Per1* and *c-Fos* in the SCN, but behavioral rhythms were delayed rather than advanced [101]. These behavioral phenotypes were only observed when light pulses were administered to mice under free-running conditions. Using an Aschoff type 2 paradigm, phase shift responses were re-evaluated in mice under LD-entrained conditions [102]. Under these conditions, *PAC1^−/−^* mice exhibited smaller phase delays and larger phase advances in response to early- and late-night light pulses, respectively [102]. These behavioral effects mirrored in vitro observations in which loss of PACAP signaling attenuated phase delays but enhanced phase advances [47]. Notably, impaired PACAP-PAC1 signaling had no effect on LD entrainment and only minor effects on SCN pacemaker activity, as reflected in a subtle shortening of the circadian period [101,103,104]. 

#### 2.1.3. Gamma-Aminobutyic Acid (GABA)

More than 90% of SCN neurons express the neurotransmitter GABA, whose effects within the central clock are mediated by GABA_A_ and GABA_B_ receptors [4,105,106,107]. GABA primarily functions to elicit spontaneous inhibitory post-synaptic potentials (IPSPs), which modulate the effects of photic inputs on SCN neurons [4,108,109,110]. The phase-dependent inhibitory effects of GABA are predominantly mediated by signaling through the chloride ion (Cl^−^) permeable ionotropic GABA_A_ receptors (GABA_A_Rs) [4,108,111,112,113,114,115], as indicated by the efficacy of the GABA_A_ antagonist, bicuculline, to block GABA- or GABA_A_ agonist-induced inhibition of SCN neuronal firing [4,114,115]. GABA_B_ receptor activation also modulates SCN neuronal activity through presynaptic inhibition of glutamate release from RHT terminals and hyperpolarization of SCN neurons [109]. The application of the GABA_B_ agonist baclofen induced a robust inhibition of SCN neurons [110], whereas co-application of GABA_A_ and GABA_B_ antagonists increased firing rates and the precision of rhythms in cultured SCN neurons [116].

GABAergic signaling has been shown to modulate non-photic phase-resetting of the central clock, both in vitro and in vivo. SCN neuronal firing rhythms were advanced or delayed by activation of GABA_A_ receptors in the mid- or late-subjective day, respectively [111,114,117]. Similarly, GABA_B_ activation by baclofen induced either a day-time phase advance or a night-time phase delay [117]. Furthermore, intra-SCN injections of the GABA_A_ agonist, muscimol, during the subjective day produced robust phase advances in hamsters [118,119].

GABA has also been demonstrated to modulate photic phase-resetting [108,118,120]. Microinjections of GABA_A_ or GABA_B_ agonists into the hamster SCN attenuated delays or advances of behavioral rhythms in response to early or late-night photic stimulation, respectively [108,118]. These effects coincided with the attenuation of light-induced *c-Fos* immunoreactivity [120]. Conversely, antagonism of the GABA_A_ and GABA_B_ receptors in the SCN augmented the phase-delaying effects of light in the early subjective night [108,120]. However, one conflicting study reported that GABA_A_ signaling is necessary for light-induced phase delays, in that sustained activation of GABA_A_ receptors in the SCN for more than 3 h resulted in phase delay of the clock [121].

GABA can exert excitatory as well as inhibitory effects [4,122,123,124,125]. This bimodality is mediated by chloride cotransporters including Na-K-Cl cotransporter 1 (NKCC1) and potassium-chloride transporter member 5 (KCC2), which determine the polarity of membrane polarization based on the relationship between the equilibrium potential of chloride and the membrane potential [123,126]. Responses to GABA via GABA_A_ receptor signaling exhibit a diurnal fluctuation which has been the subject of controversy [4,122]. Work by Wagner et al. (1997) first demonstrated that high intracellular chloride concentrations during the day facilitated excitation, whereas low nocturnal chloride concentrations facilitated inhibition [4]. However, work by De Jeu and Pennartz (2002) has challenged these findings, demonstrating instead that GABA acts as an inhibitory transmitter during the day but excites SCN neurons at night [122]. Regardless, time-of-day fluctuations in chloride concentration (and consequently, GABA sensitivity) may constitute a gating mechanism that controls the propagation of excitatory signals at different phases of the circadian cycle [122]. The polarity of responses to GABA may also be spatially organized, as a higher incidence of GABA-evoked excitation has been observed in the dorsomedial SCN [124]. Lastly, excitatory GABAergic signaling has been demonstrated to modulate photic input to the SCN, since blocking GABAergic excitatory signaling through inhibition of NKCC1 attenuated phase delays during the early subjective night [125]. 

#### 2.1.4. Serotonin (5-hydroxy-tryptamine)

The SCN is innervated by 5-hydroxytryptamine (5-HT)-containing fibers which originate from the raphe nuclei and other regions of the midbrain (Reviewed in [127]). Several 5-HT receptor subtypes have been reported to be expressed within the SCN including 5-HT_1A_, 5-HT_1B_, 5-HT_2A_, 5-HT_2C_, and 5-HT_7_ [128,129,130]. For these reasons, there has been a plethora of research investigating the modulation of the central pacemaker by 5-HT. In vivo studies have demonstrated circadian fluctuations in the levels of 5-HT in the SCN, with peak release observed at the light/dark transition and trough levels observed during the late midday [131,132]. Responsiveness to serotonergic signaling also varied according to time-of-day, reaching a peak during the nocturnal phase [133]. Single-unit recordings, both in vivo and in vitro, have demonstrated a predominantly inhibitory effect of 5-HT on SCN neurons [133,134,135,136,137]. The pharmacology of 5-HT receptor-mediated signaling within the SCN is complex. 5-HT appears to modulate glutamate release from RHT terminals via presynaptic 5-HT_1B_ and 5-HT_7_ receptors, but it also modulates GABAergic signaling via 5-HT_1B_ receptors expressed on SCN clock neurons [138,139,140,141].

It is therefore not surprising that agonists of 5-HT receptors have been shown to attenuate light-induced activity within the SCN in vivo, or to suppress optic nerve stimulation-induced excitatory postsynaptic/field potential in vitro [136,137,139,142,143,144]. At the molecular level, activation of serotonergic signaling abolished light-induced c-Fos expression in the SCN [144,145]. As shown in genetic studies, the effects of 5-HT signaling are receptor subtype-dependent [146,147,148,149]. Mice lacking the 5-HT_1A_ receptor exhibited larger phase advances and delays in response to nocturnal light exposure [148]. Light-induced *c-Fos* and *Per1* expression in the core SCN were attenuated in these animals, suggesting that the 5-HT_1A_ receptor inhibits behavioral phase shifts while facilitating light-induced gene expression in the SCN [148]. In the case of 5-HT_1B_, mice deficient for this receptor exhibited deficits in entrainment to short T-cycles and attenuated responses to light-induced phase shifts at both the behavioral and molecular level [146,147]. Although these results clearly show that 5-HT_1B_ modulates functional light input to the SCN as suggested by other studies, it is contrary to the notion that 5-HT_1B_ activation inhibits photic input [139,141,146,147]. Notably, developmental disruption of the serotonin system in mice via genetic ablation of the transcription factor *Pet-1*, which promotes serotonergic fating of neurons, resulted in attenuated early night light-induced phase delays [149,150]. However, *Pet-1* null mice also exhibited a longer free-running period, an absence of period-shortening under prolonged constant dark conditions, and a redistribution of peak locomotor activity shifting towards the late night [150]. These results indicate that serotonergic signaling regulates various circadian clock parameters and the temporal organization of activity, which may partially explain the inconsistencies in photic modulation between earlier pharmacological studies and recent studies implementing genetic models [146,147,148,149,150].

In addition to modulating photic input, the serotonergic system can have non-photic effects including phase-shifting of SCN neuronal firing, locomotor activity rhythms, melatonin secretion, body temperature rhythms, and gene expression [132,148,151,152,153,154,155,156,157,158,159,160,161,162,163,164,165]. These non-photic effects are mediated in part by the 5-HT_1A_, 5-HT_7_ and 5-HT_2C_ receptors [132,148,151,152,153,154,155,156,157,158,159,160,161,162,163,164,165]. However, there have been discordant findings on the role of serotonergic signaling. One study demonstrated that systemic application of the 5-HT_1A/7_ agonist, 7-(dipropylamino)-5,6,7,8-tetrahydronaphthalen-1-ol (8-OH-DPAT), induced phase shifts in hamsters but had no such effect on mice [163]. These results were later challenged by Horikawa and Shibata (2004), who showed that systemic administration of 8-OH-DPAT during the mid-subjective daytime induced a “clear and dose-dependent” phase advance [153]. Antle et al. (2003) also demonstrated that systemic or intra-SCN injections of the 5-HT_2A/3_ agonist, quipazine, did not significantly affect the circadian phase in either mice or hamsters, but increased or decreased, respectively, the expression of c-Fos in the SCN following daytime administration [163]. Nocturnal quipazine administration in rats induced phase advances in activity rhythms, which coincided with c-Fos induction [157]. Overall, these studies highlighted the interspecies variability in the modulation of non-photic phase shifts via serotonergic signaling, as well as the need for experimental control and consistency in technique in the field of chronobiology. Nevertheless, these studies showed that serotonin can affect circadian phase in a non-photic manner that involves multiple 5-HT subtypes, which varies across species.

Pharmacological manipulations in hypothalamic slice preparations have enabled researchers to elucidate some of the molecular underpinnings of serotonergic modulation of the SCN clock. The phase-resetting properties of 5-HT have been observed in the presence of tetrodotoxin (TTX) and high Mg^2+^, indicating that the effects of 5-HT are independent of Na^+^-induced activation of action potentials or Ca^2+^-dependent neurotransmitter release, and are likely due to direct activation of 5-HT receptors expressed on SCN neurons [159]. Furthermore, phase modulation by serotonergic stimuli is dependent on prior sensitization to serotonergic signaling, in that pre-treatment with low-dose 5-HT, 8-OH-DPAT, L-tryptophan, or fluoxetine attenuated the ability of SCN neurons to subsequently phase-shift in response to serotonergic stimulation [152]. 5-HT-induced phase advances were inhibited through the co-application of PKA inhibitors and K^+^ channel blockers, indicating that activation of PKA and K^+^ channels are necessary for 5-HT-induced phase-resetting [160]. NO signaling also contributes to the effects of 5-HT on the SCN clock, since the phase-advancing effects of 8-OH-DPAT could be attenuated by treatment with a NO synthase inhibitor or recapitulated with treatment with a NO donor [161].

#### 2.1.5. Acetylcholine

Acetylcholine (ACh) was one of the first neuromodulators postulated to play a key role in the regulation of circadian rhythms. Diurnal oscillations of acetylcholine were observed in the rodent brain, peaking during the early subjective day and declining to trough levels during the mid-subjective night [166,167]. However, ACh levels do not vary within the SCN in a time-of-day-dependent manner [168]. Despite this, cholinergic signaling via nicotinic (nAChR) and muscarinic (mAChR) acetylcholine receptors expressed by cholinoceptive neurons of the SCN has been implicated in the regulation and maintenance of circadian rhythms [169,170]. The SCN expresses messenger RNAs (mRNAs) for the mAChRs M1 to M5, and nAChR subunits A2, A3, A4, A7, and B2 [169,170,171,172,173,174]. 

Initially, cholinergic signaling was thought to modulate the effects of photic input to the circadian system [173]. Intracerebroventricular injections of carbachol, a non-selective cholinergic agonist with preferential affinity for mAChRs, evoked a time-of-day dependent phase shift in wheel running activity [173,175]. The phase-shifting effects of carbachol were also observed in slice preparations of the SCN [176,177]. However, Pauly and Horseman (1985) showed that rats treated with hemicholinium-3 to deplete ACh presynaptic stores, or atropine to block Ach receptors exhibited normal responses to light pulses, casting doubt on the notion that ACh modulates photic responses [178]. More recent work has demonstrated that cholinergic modulation of the central clock can differentially alter circadian phase by acting at multiple sites in the brain [179]. Direct application of carbachol to the SCN resulted in a non-photic-like phase advance, whereas delivery into the cerebroventricular system produced a biphasic response comparable to light [179]. These authors speculated that the photic-like effects of carbachol via ICV administration were due to stimulation of non-SCN cholinoceptive areas feeding back to the SCN to modulate phase [179]. The roles of nAChRs in non-photic phase-resetting are more elusive. Nonetheless, bath application of nicotine at most circadian times induced a robust phase advance in slice preparations, and ICV administration elicited time-of-day dependent phase shifts comparable to carbachol [174,176].

On the cellular level, the phase-resetting effects of AChRs appear to involve M1/4 mAChR signaling, in that application of the M1/4-selective agonist McN-A-343 recapitulated the “direct” phase-resetting effects of carbachol [180]. These experiments suggested that carbachol-evoked non-photic phase-shifts are in part mediated by M1/4 mAChRs [180]. Interestingly, cell-attached recordings have demonstrated that carbachol elicits both excitatory and inhibitory responses within the SCN, effects which are absent following the application of the nAChR agonists, choline and nicotine [172]. This bimodality in the action of cholinergic modulation via mAChRs in the SCN highlights a unique role of mACh signaling and may function to enable flexibility of responses under different physiological settings [172].

The molecular mechanisms mediating the effects of cholinergic signaling within the SCN have not been well elucidated. nAChRs form ligand-gated ion channels which enable the movement of cations across the cell membrane upon ACh binding. In contrast, signaling via mAChRs regulates three distinct cellular events, namely, ion conductance, breakdown of cyclic nucleotides, and the turnover of phosphatidylinositol [181,182]. In the SCN, it has been demonstrated that carbachol-evoked M1 mAChR signaling causes an influx of Ca^2+^ through the activation of phospholipase C β1 (PLCβ1) but not PLCβ4 [182]. Carbachol-induced phase-shifts also coincided with increased cGMP production and PKG activity and were inhibited by the co-administration of atropine [181]. These results demonstrated that phase modulation via ACh is at least partly mAChR-dependent and involves two distinct signal transduction pathways: the breakdown of cyclic nucleotides and phosphatidylinositol turnover [181,182].

#### 2.1.6. Glycine

In the central nervous system, glycine can function as an inhibitory neurotransmitter that binds to glycine receptor chloride ion channels (GlyRs) [183]. Glycine also works alongside glutamate as an obligatory co-agonist of NMDA receptors, facilitating NMDAR-dependent excitatory neurotransmission by potentiating receptor activation through allosteric modulation [184]. Glycinergic terminals have been found in proximity to vasopressin-expressing neurons of the SCN [185]. Furthermore, glycine release from organotypic rat SCN slice cultures exhibits time-of-day-dependent fluctuations that are similar in phase to the rhythmic release of AVP [186]. Electrophysiological experiments in dissociated or intact SCN preparations have demonstrated that glycine application can induce Cl^−^ currents that are be attenuated by the co-application of the GlyR antagonist, strychnine [73,187]. However, the duality of the actions of glycine in the SCN is evident from experiments in which co-application of glycine in the presence of glutamate enhanced NMDA-dependent glutamatergic signaling [73]. Indeed, Mordel et al. (2011) also found that glycine elicited both inhibitory and excitatory effects on SCN firing rates, with the relative proportion of each effect dependent on the circadian time [187]. In addition, a subset of SCN neurons showed biphasic responses to glycine, characterized by a rapid but transient increase in firing rate followed by prolonged suppression [187]. These findings illustrate that glycine not only functions as a classical inhibitory neurotransmitter, but also as an excitatory neuromodulator, in the central clock. 

The study by Mordel et al. (2011) provided clear evidence that glycinergic signaling can modulate SCN clock resetting [187]. Glycine application during the subjective day or early subjective night triggered a robust phase advance or delay, respectively, of neuronal firing rhythms in SCN explants [187]. Moreover, the glycine-induced phase shifts were abrogated by strychnine, indicating that they are mediated by GlyRs [187]. A more recent study by Kawai et al. (2015) suggested the glycine, through its effects on NMDA receptors, may regulate other aspects of SCN function, specifically the regulation of sleep and body temperature [188]. 

#### 2.1.7. Vasoactive Intestinal Peptide (VIP)

SCN-intrinsic neuropeptides play important roles in synchronizing SCN oscillations with the external LD cycle. VIP-expressing cells in the ventrolateral SCN receive direct innervation from the RHT [189], and must then convey photic information to the rest of the SCN. The application of VIP to ex vivo SCN slices during the late subjective night advanced the phase of neuronal firing, and stimulated the expression of *Per1* and *Per2*, mimicking the effects of light [190,191]. Consistently, mice lacking the VIP receptor, VPAC2, did not show induction of clock gene expression following a nocturnal light pulse, and exhibited impaired gating of photic responsiveness [192,193]. Moreover, entrainment to 12-h:12-h LD cycles was severely disrupted in *Vip-* and *VPAC2*-deficient mice: the observed nocturnal activity was a product of the “masking” effect of light rather than true circadian entrainment [192,194]. In the SCN, VIP-dependent phase shifts require the activity of PKA, PLC and extracellular signal-regulated kinase (ERK)/MAPK signaling pathways [190,191]. Moreover, in vitro VIP application leads to an increase in cAMP levels and a phase- and dose-dependent decrease in intracellular Ca^2+^ in the SCN [195,196]. Therefore, it is likely that VIP binding to the VPAC2 receptor in the SCN leads to cAMP-mediated activation of PKA and ERK/MAPK (Figure 1B). The VPAC2 signaling cascades converge on the activation of CRE-mediated transcription of clock genes, which confer on VIP its clock-resetting properties [190,191,195,196]. 

#### 2.1.8. Gastrin-Releasing Peptide (GRP)

A subset of ventrolateral SCN neurons expresses GRP, which has proven to be crucial for photic resetting of the clock (Figure 1A) [189,197]. GRP-positive neurons within the SCN also receive direct photic information from the retina [189,197]. GRP application to SCN slices during the early and late subjective night caused a significant delay and advance in peak neuronal firing, respectively [198]. These effects were mediated through the GRP receptor, BB_2_, as they were blocked in the presence of BB_2_ receptor antagonists [198]. In addition, in vivo microinjection of GRP into the SCN during the early and late-night potentiated phase delays and advances of locomotor activity and induced *Per1* gene expression throughout the ventral and dorsal SCN [199,200]. By signaling through its cognate G-protein coupled receptor, GRP likely employs a CREB-mediated mechanism to exert its phase-shifting effects on the clock [199,200,201]. 

### 2.2. Protein Kinases Implicated in the Regulation of Photic Entrainment of the SCN

Once photic information is conveyed to the SCN via the release of glutamate and PACAP from the RHT terminals, it triggers a series of intracellular signaling events that ultimately reset the phase of the molecular clock in SCN neurons. Various protein kinase signaling cascades are activated by photic cues and participate in the entrainment process (Figure 1B). Below we highlight some of the major protein kinases that have been implicated in the photic entrainment of the SCN. 

#### 2.2.1. Extracellular Signal-Regulated Kinases (ERK), Downstream Effector Kinases and Upstream Regulators

The p42/44 MAPK/ERK pathway serves as one of the major signal transduction pathways that couples photic stimulation to gene induction in the SCN [202]. This MAPK cascade involves the serial activation of the kinases Raf, MEK1/2, and ERK1/2 through phosphorylation by the upstream kinase. In the case of Raf, its phosphorylation is actuated by the association with the Ras GTPase. The activation of ERK1/2 is both clock-regulated and light-inducible in the SCN [202,203]. Levels of dually phosphorylated, active ERK1/2 show anti-phase oscillations in the two SCN subcompartments, peaking in the dorsal SCN in the mid to late subjective day and the ventral SCN in the early subjective night [202]. A brief light pulse during the early or late subjective night triggers a marked increase in ERK1/2 phosphorylation in the SCN [202,203]. Nocturnal light also promotes the phosphorylation of cAMP-responsive element binding protein (CREB) at Ser133 and Ser142, events that activate CREB signaling and are required for light-evoked phase shifts and gene induction in the SCN [204,205]. Activation of the MAPK pathway is required for light-induced CREB activation [206]. Phospho-active CREB triggers cAMP response element (CRE)-mediated transcription of light-responsive genes such as *Per1*, providing a mechanism by which light stimulation impinges on the TTFL to reset the clock [207]. Consistent with their roles in photic transmission, the in vitro application of glutamate and PACAP triggered an induction in p-ERK1/2 within the SCN, which is attenuated upon inhibition of NMDA receptors [94,202,203]. Moreover, pharmacological inhibition of ERK/MAPK activity caused a 50% attenuation in glutamate-dependent CREB phosphorylation, and consequently uncoupled light from clock entrainment as evidenced by the drastic suppression of light-evoked phase shifts in the SCN [208]. 

ERK1/2-dependent phosphorylation of CREB is likely to be indirect and mediated by downstream effectors of ERK1/2 signaling. p90 ribosomal S6 kinase 1 (RSK1) and mitogen- and stress-activated protein kinase 1 (MSK1) are both phosphorylated and activated by ERK1/2 [209,210]. A light pulse in the early or late subjective night, but not subjective day, triggers the phosphorylation of RSK1 and MSK1 predominantly in the ventral SCN [209,210]. Light-induced RSK1 and MSK1 activation is blocked by pretreatment with the MEK inhibitor, U0126 [209,210]. MSK1 phosphorylation in the SCN could be triggered by direct infusion of glutamate or (more potently) PACAP into the lateral ventricles of mice [210]. Conversely, infusion of a PAC1 receptor antagonist abrogated light-induced phosphorylation of MSK1 [210]. Using *msk1*-deficient mice, it was shown that absence of *msk1* led to impairments in photic entrainment: when compared to wild-type controls, *msk1^−/−^* mice were slower to re-entrain to an advanced LD schedule and displayed attenuated phase delays in response to early-night light exposure [211]. As expected, light-evoked CREB phosphorylation was reduced and *Per1* induction was nearly abolished in *msk1^−/−^* mice [211]. 

Several modulators of the ERK/MAPK signaling pathway have been described (Figure 1B) [208,212,213,214,215]. The Ras-like G-protein Dexras1 is an upstream modulator of ERK1/2 activity in the SCN [212]. *Dexras1^−/−^* mice exhibit an unusual photic phase response curve (PRC), reacting to late-night but also mid-day light pulses with large phase advances, and showing smaller phase delays in response to early-night light stimulation [213]. Whereas the diminished phase delay was correlated with reduced ERK1/2 activation in the ventral SCN of *dexras1^−/−^* mice [212], the phase advance caused by late-night light exposure was associated with a significant augmentation of light-induced p-ERK1/2 expression, which was unusually distributed across the entire SCN [213]. This latter effect was shown to be dependent on PACAP and was abolished upon pharmacological inhibition of either MEK or PACAP signaling [213]. Further in vitro and in vivo analyses demonstrated that Dexras1 serves as a negative regulator of ERK1/2 activation by PACAP, and that it acts as a gate to restrict daytime access of photic information to the SCN [213]. 

The Raf kinase inhibitor protein (RKIP) is another modulator of MAPK signaling [216]. Nocturnal light pulses trigger the phosphorylation of RKIP at Thr-153, resulting in its dissociation from and derepression of Raf [214]. Expression of the phosphorylation-deficient RKIP (T153V) mutant, which constitutively inhibits Raf, in the SCN blocked early-night light-evoked ERK1/2 activation and phase delays [214]. In contrast, *RKIP^−/−^* mice showed significantly larger phase delays and advances in response to early- and late-night photic stimulation, respectively [214]. Consistent with the augmented phase shifts, *RKIP^−/−^* mice exhibited prolonged ERK1/2 activation in the SCN following nocturnal light exposure, in addition to enhanced transcription of *Per1* and *c-Fos* [214]. Ca^2+^/calmodulin-dependent protein kinase (CaMK) signaling has also been shown to be an upstream modulator of light-induced ERK1/2 activity in the SCN, ultimately affecting behavioral phase shifts and clock gene induction [208,215].

#### 2.2.2. Mammalian Target of Rapamycin (mTOR)

Mammalian target of rapamycin (mTOR) is downstream of several prominent protein kinases including the MAPK/ERK signaling cascade. A member of the phosphatidylinositol 3-kinase (PI3K)-related protein kinase family, mTOR is a major component of two distinct complexes, mTOR complexes 1 (mTORC1) and 2 (mTORC2), that regulate diverse cellular processes. In particular, mTORC1 plays a prominent role in regulating protein synthesis or translation. mTORC1 signaling is mediated by two distinct downstream effectors: p70 S6 kinase (p70S6K) and eukaryotic translation initiation factor 4E (eIF4E)-binding protein 1 (4E-BP1). P70S6K phosphorylates S6 ribosomal protein to stimulate translation of mRNAs with a 5′-terminal oligopyrimidine tract (5′TOP mRNAs), whereas 4E-BP1 inhibits eIF4E-mediated cap-dependent translation initiation. Phosphorylation by mTORC1 activates p70S6K and inhibits 4E-BP1. 

Similar to ERK1/2, mTOR signaling is under both circadian and photic regulation in the SCN [217,218]. Levels of phospho-S6 within the SCN are rhythmic, with peak and trough expression observed in the subjective day and night, respectively [218]. Light exposure in the subjective night, but not subjective day, triggers the phosphorylation of mTOR at Ser2448 (a marker of mTOR activity), p70S6K, 4E-BP1, and S6 ribosomal protein in the murine SCN [217,219]. Infusion of rapamycin (mTOR inhibitor) or U0126 abrogated light-evoked phosphorylation of p70S6K and S6, placing them downstream of mTOR and MAPK/ERK signaling, respectively [217]. Furthermore, light-induced phosphorylation of p70S6K and S6 in the SCN was strongly suppressed by infusion of antagonists of PAC1 receptors or ionotropic glutamate receptors, whereas S6 phosphorylation was triggered by infusion of PACAP and glutamate [219]. This indicates that mTOR/p70S6K signaling is downstream of PACAP and glutamate neurotransmission [219]. Importantly, the clock resetting effects of light were sensitive to rapamycin, such that early-night phase delays were attenuated, but late-night phase advances were potentiated, by infusion of rapamycin [219]. Rapamycin attenuated the induction of PER1 and PER2 in the SCN in response to early-night light pulses, an effect that was not sensitive to actinomycin D (a transcription inhibitor), suggesting that the underlying mechanism is post-transcriptional [219]. In line with a possible translational mechanism, the expression of eukaryotic elongation factor 1A (eEF1A), which is encoded by a 5′-TOP mRNA, within the SCN is both light-inducible and rapamycin-sensitive [219]. Subsequent mouse knockout studies provided additional evidence supporting a role of mTOR signaling in photic entrainment [220]. Mice genetically deficient for 4E-BP1 (*eif4ebp1^−/−^*) exhibited accelerated re-entrainment to shifted LD cycles and were more resistant to constant light-induced behavioral arrhythmicity [220]. In the absence of 4E-BP1, there was greater expression of VIP in the SCN due to enhanced translation initiation [220]. The rapid LD re-entrainment of *eif4ebp1^−/−^* mice was rescued by infusion of a VPAC2 antagonist into the SCN [220]. Importantly, the *eif4ebp1^−/−^* phenotype was consistent with the phenotype of mice carrying a single functional copy of the *mtor* gene (*mtor^+/−^*), which were more susceptible to LL-induced arrhythmicity and expressed lower abundance of VIP [220]. 

#### 2.2.3. c-Jun NH2-Terminal Kinases (JNK)

The c-Jun NH2-terminal kinase (JNK) cascade represents another major MAPK signaling module. The terminal kinase, JNK, is phosphorylated and activated by the MAPK kinases, MKK4 and MKK7, which are in turn activated by their respective upstream kinases. Rhythmic phosphorylation of JNK1 in the hamster SCN was first reported by Pizzio et al. (2003) [221]. Phospho-JNK1 oscillations peaked under LD and DD conditions at ZT 12 and CT 4, respectively, although it is not clear why the oscillatory phase differed so markedly between the two conditions [221]. JNK1 phosphorylation in the SCN was inducible by nocturnal (but not daytime) photic stimulation [221]. To examine the role of JNKs in central timekeeping, Yoshitane et al. (2012) characterized the circadian phenotype of *jnk3*-deficient mice, as *jnk1*/*jnk2* double nullizygous animals were embryonic lethal [222]. The expression of JNK3 is restricted to the nervous system, and its ablation resulted in animals with reduced clock-resetting capacity in response to early and late-night photic stimulation [222]. The behavioral phase shift phenotype could not be attributed to deficiencies in photic induction of *Per1*, which remained unaltered in these animals compared to controls [222]. Interestingly, *jnk3^−/−^* mice had a much longer free-running period under DD, but did not display the period-shortening effects of late-night photic stimulation that control animals showed [222]. The absence of this “aftereffect” to light suggests that *jnk3^−/−^* mice do not adapt appropriately to prior photic experience. This is further reflected in their behavior under LL, in which these mutant animals did not exhibit the expected period lengthening in response to increasing light intensity [222]. The underlying mechanisms for the *jnk3*-null phenotype remain unclear.

#### 2.2.4. p38 MAPK

In contrast to the ERK or JNK pathways, there is limited evidence on the role of the p38 MAPK pathway in central circadian timekeeping in mammals. Pizzio et al. (2003) reported rhythmic p38 phosphorylation in the hamster SCN under LD and DD conditions, peaking at ZT/CT 8 [221]. A brief light pulse in the middle of the subjective night, but not in the subjective day, induced p38 phosphorylation [221]. To date, there are no gene knockout studies to examine the significance of p38 to photic entrainment or central pacemaking. 

#### 2.2.5. Ca^2+^/Calmodulin-Dependent Protein Kinase II (CaMKII)

Various studies have implicated Ca^2+^/calmodulin-dependent protein kinase II (CaMKII) in the photic entrainment process. There are 4 known isoforms of CaMKII (α, β, γ, δ), with the δ isoform being the predominant form in the rat SCN [223]. The phosphorylation of CaMKII is both rhythmic, peaking in the subjective day, and inducible by nocturnal light in the ventral SCN of hamsters [215,224]. Central administration of pharmacological inhibitors of CaMKII have been shown to attenuate light-induced phase delays and advances in hamsters [215,225]. In line with these effects, CaMKII or calmodulin antagonists attenuated glutamate-induced delays in SCN firing rates ex vivo, and systemic delivery of calmodulin antagonists suppressed light-evoked expression of c-Fos in the rat SCN [226]. CaMKII inhibition has also been demonstrated to attenuate light-induce *Per1* and *Per2* expression in the hamster SCN [215]. The *mPer1* promoter region bears elements that are responsive to CaMKIIδ in in vitro assays [223]. Moreover, the pharmacological inhibition of CaMKII suppressed light-induced MAPK/ERK activation in the murine SCN, placing CaMKII upstream of MAPK/ERK signaling following photic stimulation [208]. 

#### 2.2.6. cAMP-Activated Protein Kinase (PKA)

cAMP-activated protein kinase (PKA) is a major effector of cAMP signaling, leading to the phosphorylation and activation of CREB. Many of the initial studies utilized ex vivo SCN electrical rhythms to examine the contribution of PKA signaling to clock resetting [87,191,227]. Tischkau et al. (2000) reported that application of the PKA inhibitor, KT5720, suppressed glutamate-induced phase delays of electrical rhythms in the early night but potentiated glutamate-induced advances in the late night [87]. Glutamate-triggered *Per1* induction in the early, but not late, night was abolished by KT5720, suggesting that PKA is required for coupling photic-like signals to the molecular clock during the early night [87]. In addition to glutamate, the effects of VIP appear to be mediated in part by PKA [191,228]. VIP- or VPAC2 agonist-induced phase advances of SCN firing rhythms in the late night could be abrogated by pre-treatment with KT5720 [191]. Interestingly, brief stimulation of SCN slices with VIP during the subjective night resulted in a persistent increase in the excitability of dorsal SCN neurons, an effect that was greatly diminished by the PKA inhibitor, H89 [228]. Furthermore, tonic PKA activity appears to play a role in setting the phase of the SCN clock: application of the PKA inhibitor, Rp-cAMPS, in the late day (CT 10) delayed SCN electrical rhythms by approximately 2h, with no effect being observed when the inhibitor was applied at other times of day [227]. As cAMP levels are known to fluctuate in the SCN, peaking in the mid-day and returning to trough levels at the day-to-night transition, it suggests that PKA activity during this critical period—the late day—is important for setting the circadian phase [229]. The in vivo effects of PKA activity on clock resetting were recently examined by Sterniczuk et al. (2014) using a hamster model [230]. Light-evoked phase delays in the early night were attenuated by infusion of KT5720 into the third ventricle [230]. KT5720 similarly diminished phase delays induced by GRP infusion in the early night, but GRP-induced phase advances in the late night were unaffected [230]. 

#### 2.2.7. Protein Kinase C (PKC)

Protein kinase C (PKC) has also been implicated in photic entrainment of the SCN [231,232,233]. Isoforms of PKC are classified as conventional (α, β, γ), novel (δ, ε, η, θ), or atypical (ζ, ι, λ) based on whether they require Ca^2+^ and diacylglycerol (DAG), DAG but not Ca^2+^, or neither, respectively, for their activation. A nocturnal light pulse induces the phosphorylation and activation of PKC in the murine SCN [233]. Central administration of the pan-PKC inhibitor, bisindolylmaleimide I, augmented the phase-delaying effects of early night light exposure and suppressed light-evoked PER1 (but not PER2) expression in the SCN by enhancing PER1 degradation [233]. This suggests that PKC is a negative regulator of photic entrainment. On the other hand, the α isoform of PKC was suggested to positively modulate the clock-resetting effects of light [232]. Conventional PKCα (*Prkca*) knockout mice showed a pronounced reduction in early-night light-induced phase delays with no alteration in clock gene induction, thereby ruling out CRE-mediated transcriptional activation as a potential mechanism [232]. Rather, it was demonstrated that, upon photic stimulation, PKCα physically interacted with and phosphorylated the clock protein PER2 in SCN neurons, protecting it from degradation and retaining it in the cytoplasm [232]. Given that induction of PER2 is essential for light-induced phase delays [234,235], the attenuated phase delays exhibited by *Prkca^−/−^* mice were attributed to the greater destabilization of PER2 protein following a light pulse [232]. Systemic delivery of the broad-spectrum PKC inhibitor, NPC-15437, also attenuated both light-induced phase delays and advances [231]. Collectively, these studies suggest that PKC may have isoform-specific effects on photic entrainment, or that PKC may be acting in other tissues or brain regions to differentially modulate the entrainment process. 

#### 2.2.8. cGMP-Dependent Protein Kinase (PKG)

Several studies have implicated cGMP/PKG signaling in the photic resetting specifically during the latter half of the subjective night. Infusion of PKG inhibitors, including KT-5823, into the third ventricle of hamsters strongly attenuated light-induced advances but not delays of behavioral rhythms [236,237]. Inhibitors of guanylyl cyclase (GC), which block the production of cGMP, were shown to have a similar suppressive effect on light-evoked phase advances, whereas sildenafil (also known as Viagra), a cGMP phosphodiesterase (PDE) inhibitor that blocks the hydrolysis of cGMP, potentiated phase advances without affecting delays in hamster models [238,239]. In addition, systemic administration of sildenafil in the mid-subjective night dose-dependently accelerated re-entrainment to an advance of the LD schedule [239]. Both the rat and hamster SCN exhibit a circadian oscillation in cGMP levels, which peaks for ~4 h in the late subjective night to early subjective morning and is likely mediated by the rhythmic variation in the activity of cGMP PDE but not GC [88,238]. Consistent with this, rhythmic PKG activity in the rat and hamster SCN also peaks in the late night/early morning [88,238]. A phase-advancing but not -delaying light pulse triggers a rise in cGMP levels in the SCN, which is consistent with the notion that cGMP/PKG mediates the phase-shifting effects of photic stimulation in the late night [238]. Mathur et al. (1996) reported that infusion of cGMP agonists into the hamster SCN had no effect on the phase of behavioral rhythms, although Liu et al. (1997) showed that the firing rhythms of rat SCN ex vivo could be phase-advanced by the cGMP agonist, 8-Br-cGMP, applied during the night [181,237]. It would appear that the rise in endogenous cGMP levels during the late night may be functionally significant to the state of the clock, as KT5823-mediated inhibition of PKG in the late night phase-delays various clock outputs, including rhythms in locomotor activity, SCN neuronal firing, cGMP levels, and *Per1* mRNA expression [88]. The phase-resetting effects of KT5823 on SCN electrical rhythms are rapid, with delays seeming to occur in stepwise increments depending on the duration of KT5823 treatment [88]. Moreover, transiently knocking down PKG-II, the major form of PKG in the rat SCN, in the mid-to-late night using PKGII-specific antisense oligodeoxynucleotides (ODN) also induces delays of SCN electrical rhythms, whereas chronic ODN treatment disrupts these rhythms [88]. Although it is not entirely clear how light couples to the increase in cGMP production and PKG activation, there is some evidence to suggest that cholinergic signaling induces both, and that both cGMP and PKG are required for the in vitro phase-advancing effects of carbachol [181]. 

#### 2.2.9. Salt-Inducible Kinases (SIKs)

Salt-inducible kinases (SIKs) belong to the 5′-AMP activated protein kinase (AMPK) family of proteins that serve as energy sensors and regulate metabolism. SIK1 was identified by Jagannath et al. (2013) in a screen for light-inducible genes in the SCN [240]. *Sik1* is a CRE-regulated gene that phosphorylates and induces the degradation of the CREB co-activator, CREB-regulated transcription coactivator 1 (CRTC1) [240,241]. Interestingly, the abundance of CRTC1 protein in the SCN is rhythmic, exhibiting a day-time peak and a night-time nadir, and a nocturnal light pulse induces its rapid accumulation in the nucleus [242]. Infusion of *sik1*-specific siRNAs into the third ventricle of mice resulted in enhanced phase delays in response to early-night photic stimulation, as well as more rapid re-entrainment to an advance of the LD schedule [240]. *Sik1* knockdown also elicited a modest but significant increase in behavioral period under DD conditions [240]. The findings suggest that SIK1 provides feedback inhibition to the SCN in response to photic signals, limiting the clock-resetting effects of light on the central pacemaker. 

More recently, SIK3 was shown to play an important role in circadian entrainment [243]. *Sik3* transcripts are expressed in the murine SCN in a non-oscillatory manner [243]. *Sik3*-deficient mice exhibited large (~6 h) phase delays in the rhythms of various clock outputs, including oxygen consumption, rectal temperature, and food consumption [243]. In addition, locomotor activity rhythms were poorly entrained to the external LD schedule, displaying variable and unstable onsets as well as weakly consolidated activity [243]. *Sik3^−/−^* mice free-ran with a significantly longer period in DD than control mice and were much slower to re-entrain to a new LD schedule imposed after the DD interval [243]. SCN slices cultured ex vivo exhibited desynchronized molecular rhythms with disperse periods and phases [243]. In vitro assays revealed that overexpression and knockdown of SIK3 decreased or increased, respectively, the stability of PER2 [243]. Lastly, Hayasaka et al. (2017) showed that SIK3 directly phosphorylates PER2 and promotes its degradation in a manner that is insensitive to casein kinase 1 (CK1) inhibitors, suggesting that the two kinases mediate independent phosphorylation events on PER2 to control its stability [243]. 

#### 2.2.10. Casein Kinase 1 (CK1)

Casein kinase 1 epsilon (CK1ε) is the first mammalian protein kinase implicated in period length determination but was recently shown to play a role photic entrainment [244,245,246]. CK1ε is one of seven known isoforms of mammalian CK1 (α, β1, γ1, γ2, γ3, δ, and ε) and has been demonstrated to phosphorylate PER1 and PER2, promoting their degradation [245]. CK1ε-deficient mice exhibited faster re-entrainment to an advance or delay of the LD schedule, as well as potentiated phase delays and advances in response to nocturnal light pulses [246]. NMDA-induced phase shifts of PER2::LUCIFERASE (PER2::LUC) bioluminescence rhythms were also enhanced in CK1ε-deficient SCN explants [246]. The resistance of these mutant mice to jetlag was further exemplified by reduced desynchrony between physiological rhythms, such as body temperature and locomotor activity, when the LD schedule was shifted [246]. Pharmacological inhibition of CK1ε mimicked the in vivo and ex vivo effects of genetic ablation [246]. The study suggested that a key function of CK1ε is to buffer the SCN clock against phase shifts by limiting light-induced PER accumulation [246].

#### 2.2.11. Glycogen Synthase Kinase 3 (GSK3)

Beyond its well-described role in period length determination, glycogen synthase kinase 3 (GSK3) has recently been implicated in photic entrainment of the SCN [247]. There are two GSK3 genes encoded in the mammalian genome: GSK3α and GSK3β. The activity of GSK3β is induced by light, as indicated by the dephosphorylation of GSK3β at Ser9 (a prerequisite for GSK3β activation) in the whole SCN following late-night light exposure [247]. Pharmacological inhibition of GSK3 abrogated the persistent increase in the electrical activity of the SCN in response to a late-night light pulse [247]. Using constitutively active GSK3 knock-in mice in which both the α and β isoforms were rendered phosphorylation-deficient, Paul et al. (2017) revealed that constitutive GSK3 activity accelerated the rate of re-entrainment to an advanced, but not delayed, LD schedule, an effect that was not due to changes in non-circadian light processing [247]. However, acute clock resetting to a late-night light pulse was not affected in these animals [247]. Furthermore, mice expressing constitutively active GSK3 exhibited an advanced phase angle of entrainment, which was mirrored by the advanced phase of light-evoked increase in SCN excitability [247]. These collective observations indicate that GSK3 plays an important role in photic entrainment at the behavioral and neurophysiological level. 

#### 2.2.12. G-protein Coupled Receptor Kinase 2 (GRK2)

G-protein coupled receptor kinases (GRKs) are known to phosphorylate activated GPCRs and induce their desensitization, internalization, and/or degradation. The mammalian genome encodes 7 GRKs (GRK 1-7), and GRK2 was recently demonstrated to modulate both the entrainment and the intrinsic circadian properties of the SCN [248]. *Grk2* deficiency in mice enhanced light-induced phase delays, suppressed phase advances, and slowed the rate of re-entrainment to an advanced LD schedule [248]. In addition, free-running period under DD and LL conditions were markedly longer in *grk2*-deficient mice [248]. The potentiated phase delays correlated with augmented light-evoked ERK1/2 activation and *Per1* induction in the SCN, whereas the lengthened free-running rhythms correlated with heightened amplitude of PER1 and PER2 cycling in the SCN [248]. Unexpectedly, *grk2*-deficient SCN showed greater nuclear accumulation of PER1 and PER2, and overexpression of GRK2 in cultured cells promoted cytoplasmic retention of PER1 and PER2 [248]. In vitro analyses identified Ser-545 on PER2 as a potential site of phosphorylation by GRK2 [248]. These findings suggest that GRK2 regulates photic entrainment through a canonical mechanism that involves modulation of GPCR-MAPK signaling, but that it may influence the pace of the molecular clock by modulating the nuclear trafficking and accumulation of PER proteins in a kinase-dependent manner [248]. 

## 3. Neuromodulation of Circadian Timekeeping and Synchrony within the SCN

### 3.1. The Role of Neurotransmitters and Neuropeptides

#### 3.1.1. GABA

GABA has been implicated in the establishment of synchrony of circadian oscillators. Application of GABA on dissociated SCN neuronal cultures induced synchronization of clock cells through activation of GABA_A_ receptors [114]. Furthermore, the state of the SCN network has been shown to dictate the effects of GABA acting on GABA_A_ receptors [249]. GABA_A_ signaling promotes synchrony when the network is in an antiphase configuration, but opposes synchrony under steady-state conditions, thereby providing a mechanism that is purported to enable the fine-tuning of phase relationships according to physiological conditions [249,250]. One of these physiological conditions may be season-dependent changes in daylength [251,252]. Recent work has demonstrated that seasonal time is encoded by the phase reorganization between the dorsal and ventral SCN [251,252]. These adjustments to phase are mediated by changes in the concentration of intracellular chloride, which can alter the strength and polarity of GABA_A_ signaling [251,252].

Recent work by Barca-Mayo et al. (2017) highlighted the role of SCN astrocytic GABAergic signaling in the modulation of circadian rhythms [253]. The authors demonstrated that ablation of the clock gene *Bmal1* in astrocytes induced a bimodal pattern of locomotor behavior arising from perturbations of the neuronal clock [253]. Changes in neuronal clock gene expression were driven by an over-inhibition caused by increased GABA release from SCN astrocytes [253]. Treatment with a GABA_A_R antagonist rescued the locomotor desynchrony in these animals, indicating that GABA_A_ signaling mediates astrocyte-to-neuron communication within the SCN [253]. 

#### 3.1.2. VIP

Various in vivo and in vitro studies have uncovered mechanisms by which the SCN achieves network coherence, with most implicating signaling via VIP and AVP. For example, *VPAC2^−/−^* mice entrained significantly faster to experimental jetlag and showed markedly damped circadian amplitude of clock gene expression [192]. Analysis of electrical activity of dispersed SCN neurons and organotypic cultures showed that only 30% of *VPAC2-* or *Vip*-null neurons maintained rhythmic firing compared to 70% rhythmicity observed in wild-type neurons [194]. Those *VPAC2^−/−^* or *Vip^−/−^* neurons that remained rhythmic nevertheless displayed low amplitude rhythms, broad distribution in period lengths, and random phase relationships relative to one another, indicating that their oscillations were desynchronized [194,254]. Application of a VPAC2 receptor antagonist significantly reduced the number of oscillating neurons in *Vip^−/−^* SCN cultures, suggesting that residual VPAC2 signaling contributed to the low proportion of rhythmic *Vip^−/−^* neurons [254]. Importantly, synchrony among *Vip^−/−^* SCN neurons was restored upon the application of a VPAC2 receptor agonist [194]. 

As expected, absence of VIP-VPAC2 signaling resulted in erratic behavior under constant dark (DD) conditions [192,194]. *VPAC2^−/−^* and *Vip^−/−^* mice displayed low-amplitude rhythms, random activity onsets, low activity counts, and multiple free-running periods of locomotor activity rhythms when released into DD, all of which are suggestive of a dysfunctional clock and consistent with molecular data from the SCN of these mice [192,194]. However, constant light (LL) appeared to mitigate the loss of VIPergic signaling observed in *VPAC2^−/−^* mice [255]. Under LL, there was a remarkable improvement in behavioral rhythmicity, an effect attributed to enhanced synchrony in molecular oscillations of the ventral and dorsal regions of the SCN [255]. 

The circadian phenotypes of *VPAC2^−/−^* and *Vip^−/−^* mice suggest that the loss of VIP signaling imposes a state of damped oscillation and desynchrony within the SCN network, which is beneficial under conditions of forced desynchrony/jetlag but is detrimental to the oscillatory activity of the SCN as a whole under steady-state conditions. Interestingly, a role for VIP in reducing intercellular synchrony has been reported [256,257]. When applied to SCN explants, VIP at concentrations above 100nM attenuated oscillations in PER2::LUC expression [256]. This is primarily due to the “phase tumbling” effect, where VIP in high concentrations causes unpredictable shifts in the phase of individual cell oscillations leading to low-amplitude rhythms at the population level. Consistent with this explanation, mice that received microinjections of VIP into the SCN displayed accelerated re-entrainment to an advanced LD schedule, phenocopying the behavior of *VPAC2^−/−^* and *Vip^−/−^* mice [256]. Similarly, in vitro SCN explants treated with 10 µM VIP showed rapid entrainment of PER2 rhythms to phase shifts in temperature cycles [256]. Computational modeling predicted that coupling via VIP is only achieved if its temporal release coincides with the time of peak activity in *Per* expression, i.e., during the circadian day [257]. 

Apart from shielding the SCN against environmental perturbations, interneuronal coupling is indispensable for resistance of the SCN against genetic mutations [258,259]. When a “host” *Vip^−/−^* organotypic SCN slice was cultured in the presence of a *Vip^+/+^* SCN “graft”, an immediate and significant enhancement of circadian amplitude and synchrony was observed in PER2::LUC oscillations of the *Vip^−/−^* SCN host, regardless of the spatial orientation of the graft relative to the host tissue [259]. These results signify a role of paracrine signaling in SCN neuronal coupling, and negate the requirement for direct, synaptic communication. When *VPAC2^−/−^* SCN host tissues were co-cultured with *VPAC2^+/+^* SCN grafts, a similar but delayed restoration of amplitude and synchrony was observed [259]. However, this restoration was suppressed by pharmacological inhibition of GRP- or AVP-ergic signaling, further supporting the compensatory roles of diverse signaling factors in the preservation of robust SCN oscillations [259]. Such paracrine factors are also crucial in sustaining rhythmicity in the presence of mutations of core clock genes [258,259]. Coherent, high-amplitude oscillations in PER2::LUC rhythms could be restored in arrhythmic *Cry1^−/−^Cry2^−/−^* SCN slices by co-culturing them with wild-type SCN tissues [259]. 

#### 3.1.3. Arginine Vasopressin (AVP)

The *Avp* gene is clock-controlled and rhythmic by virtue of E-box motifs within its promoter [50]. Mice genetically deficient for the AVP receptors, V1a and V1b, exhibited rapid re-entrainment to advanced and delayed LD schedules, a phenotype that is explained by a weak oscillator that is highly susceptible to fluctuations in light conditions and thus resistant to jetlag [260]. In line with the behavior, an arrhythmic pattern of clock gene expression was observed in both wild-type and *V1a^–/–^V1b^–/–^* SCN immediately following an LD advance [260]. However, recovery to high-amplitude oscillations was markedly accelerated in *V1a^–/–^V1b^–/–^* mice compared to wild-type animals, which is consistent with their rapid re-entrainment [260]. Individual cell recordings of *Per1*-luc bioluminescence in SCN explants indicated that, upon clock resetting, oscillations in *V1a^–/–^V1b^–/–^* cultures showed an immediate loss of phase coherence, suggesting an impairment in synchrony [260]. However, unlike the *VPAC2^−/−^* mice, *V1a^–/–^V1b^−/−^* mice had no deficits in phase shifts or clock gene induction in response to brief light pulses, and had normal behavior and expression of clock genes under standard LD and free-running conditions [260]. These findings illustrate the importance of AVP-V1a/b signaling in interneuronal coupling and SCN synchrony, which ultimately impacts entrainment to environmental light cycles. 

Other mouse models in which clock mutations were restricted to AVPergic neurons showed a similar resistance to jetlag, along with other perturbations [261,262]. Ablation of *Bmal1* in AVP-positive SCN neurons promoted the uncoupling of the morning and evening peaks of behavior, reminiscent of the “splitting” behavior observed in hamsters and the likely consequence of uncoupling of individual SCN oscillators [261]. There was significant attenuation of the expression of critical clock genes such as *Per1*, *Avp*, and *Prokineticin 2* in the dorsal SCN of these animals [261]. In addition, PER2::LUC oscillations specifically in dorsal SCN neurons of these mutant mice exhibited reduced amplitude and period instability [261]. 

Similarly, mouse models harboring genetic ablation or overexpression of *casein kinase 1 delta (CK1δ*) in AVPergic neurons exhibited lengthened and shortened free-running period, respectively [262]. This was observed not only at the behavioral level but also at the level of cellular period in dorsal SCN neurons [262]. However, in vitro assays demonstrated that the period elongation in PER2::LUC rhythms in *Avp-CK1δ^−/−^* SCN explants was transient [262]. Surgical disruption of core-to-shell communication resulted in sustained period elongation in PER2::LUC rhythms in the dorsal SCN of *Avp-CK1δ^−/−^* mice [262]. *Avp-CK1δ^−/−^* mice re-entrained significantly faster to an 8h advance of the LD schedule compared to wild-type controls, but this was achieved by phase delays rather than phase advances [262]. Together, the studies by Mieda et al. (2015, 2016) suggest that AVP clock neurons within the SCN are critical determinants of SCN entrainment and intrinsic circadian pacemaking function [261,262]. 

#### 3.1.4. GRP

The work of Maywood et al. (2006, 2011) demonstrated the importance of GRP to cellular synchrony within the SCN network [259,263]. Although pharmacological inhibition of BB_2_ receptors did not affect the rhythmicity of wild-type SCN explants, exogenous application of GRP transiently restored rhythmicity and cellular synchrony in *VPAC2^−/−^* SCN slices [263]. Along these lines, BB_2_ antagonists abolished the ability of a wild-type SCN graft to restore rhythmicity to a VPAC2*^−/−^* SCN host [259]. These studies suggest that although GRP-BB_2_ signaling is dispensable for synchrony in the wild-type SCN, it becomes a limiting paracrine factor that supports and is essential for SCN synchrony in the absence of VIP-VPAC2 signaling [259,263]. 

#### 3.1.5. Prokineticin 2

Prokineticin 2 (Prok2 or PK2) and its cognate G-protein coupled receptor, prokineticin receptor 2 (Prokr2), are highly expressed in the SCN and influence its activity [264,265,266,267]. The *Prok2* gene harbors E-box elements in its promoter, rendering it a clock-controlled gene: in the SCN, rhythmic *Prok2* expression exhibits a daytime peak, and is severely perturbed in *Clk* and *Cry* mutant mice [264]. Early- and late-night light pulses induced the expression of *Prok2* mRNA, and phase-shifted its rhythmic profile; however, despite its light-regulated nature, PK2 signaling was shown to be dispensable for SCN entrainment [264,266]. *Prok2^−/−^* and *Prokr2^−/−^* mice displayed rhythmic behavior, albeit with significantly reduced circadian amplitude and a drastic decrease in wheel-running activity levels [265,266]. These results are at odds with the observation that ICV injections of recombinant PK2 suppressed nocturnal activity levels [264], a discrepancy that may be explained by PK2 concentrations, the site of action, or compensatory effects in the *Prok2^−/−^* mouse model. *Prok2^−/−^* and *Prokr2^−/−^* mice showed an unusual distribution of wheel-running behavior, in which the majority of their nocturnal activity was confined to the late night as opposed to the early night [265,266]. The effects of *Prok2* or *Prokr2* ablation on circadian amplitude and activity patterns likely stem from perturbations that are downstream of the molecular clockwork, since clock gene expression was not affected in the SCN of *Prok2^−/−^* or *Prokr2^−/−^* mice [265,266]. However, overexpression of PK2 caused a slight but significant reduction in transcript levels of *Bmal1*, *Per1*, *Per2*, and *Cry2* in the SCN [267]. Thus, unlike some of the neuropeptides described above, PK2 signaling is required for the magnitude and consolidation of rhythmic behavior, but it has no effect on entrainment of the SCN and only minor effects on circadian pacemaking [265,266,267].

### 3.2. Protein Kinases that Modulate Circadian Period

Although many protein kinases have been implicated in photic entrainment through their participation in various light-triggered signaling cascades, far fewer have been shown to regulate the pace of the molecular clock. Notwithstanding, three families of protein kinases—CK1, CK2, and GSK3—have well-described effects on circadian period, through the phosphorylation of PER2 and other core clock proteins (Figure 1B). Generally speaking, it is unclear whether receptor-mediated signaling events can also impinge on these kinases to couple environmental cycles to persistent changes in circadian period.

#### 3.2.1. CK1

A semi-dominant mutation in CK1ε was the first clock mutation identified in mammals: hamsters bearing one or two copies of this natural mutation, *tau*, exhibited a 2h or 4h shortening, respectively, of the circadian period [244]. However, mice deficient for the wild-type version of CK1ε displayed either no or modest period lengthening, as reflected in their locomotor activity rhythms and PER2::LUC bioluminescence rhythms in neonatal SCN explants [268,269]. In addition to CK1ε, the rodent SCN also expresses CK1δ: both isoforms are expressed at constant levels throughout the 24h cycle and exhibit delayed induction (at the mRNA level) in response to nocturnal light stimuli [270,271]. The period of PER2::LUC rhythms was markedly longer in SCN explants prepared from CK1δ-deficient neonatal mice compared to wild-type controls [269]. The role of CK1δ in period length determination was further demonstrated in a pharmacological study that compared the relative importance of CK1δ and CK1ε [272]. A CK1δ-selective inhibitor, PF-670462, evoked dose-dependent period lengthening of PER2::LUC rhythms in SCN explants, whereas a CK1ε-selective inhibitor did not [272]. Furthermore, systemic administration of PF-670462 lengthened behavioral period in wild-type but also CK1ε-deficient mice, whereas the CK1ε inhibitor elicited period lengthening only in mice expressing the *tau* mutant (gain-of-function) form of CK1ε and not wild-type animals [272]. These studies indicate that CK1δ, not CK1ε, has a dominant role in defining the circadian period. The effect on period length is due to the phosphorylation-dependent degradation of PER proteins. CK1δ has been shown to phosphorylate PER1 and PER2, and is sufficient for the phosphorylation of PER2 at Ser659 [271,273]. Initial Ser659 phosphorylation is required for subsequent phosphorylation of downstream serine residues: thus, CK1 serves as the priming kinase for phosphorylation and degradation of PER2 [273]. In line with this hypothesis, systemic administration of PF-670462 promoted nuclear retention of PER2 in the SCN in the subjective night, thereby extending the duration of PER2-mediated feedback inhibition of the molecular clock [272]. 

#### 3.2.2. Casein Kinase 2 (CK2)

Several studies have implicated mammalian casein kinase 2 (CK2) in circadian pacemaking. A CK2 tetramer is comprised of two catalytic α subunits and two regulatory β subunits; however, the β subunit is not necessary for the function of the α subunit. Using an RNA_i_ based screen, Maier et al. (2009) identified CK2 as an important period-determining kinase [274]. Knockdown of CK2α, CK2α’, or CK2β resulted in significant period lengthening in human osteosarcoma U2OS cells expressing a *Bmal1*-luciferase reporter, whereas overexpression of CK2α/α’ induced period shortening [274]. Similarly, pharmacological inhibition of CK2 lengthened the period of PER2::LUC rhythms in SCN explants and reduced the oscillatory amplitude [275]. Both studies found that CK2 physically associates with and phosphorylates PER2, although the outcome on PER2 stability is debatable [274,275]. Maier et al. (2009) showed that CK2 phosphorylated PER2 at N-terminal residues (between residues 10 and 15), and CK2 inhibition led to reduced accumulation of PER2 in the cytosol and nucleus [274]. On the other hand, Tsuchiya et al. (2009) reported a different CK2-targeted phosphorylation site on PER2 (Ser53), and showed that CK2 inhibition or CK2α overexpression suppressed or enhanced, respectively, the degradation of PER2 [275]. CK2α was also reported to phosphorylate *Bmal1* at Ser90 to promote its entry into the nucleus [276]. 

#### 3.2.3. GSK3

Pharmacological and genetic studies have provided strong evidence supporting a role of GSK3 in period length determination in mammals. Mice that were fed rodent chow containing lithium, a potent inhibitor of GSK3, displayed significantly longer free-running locomotor rhythms [277]. Both GSK3α and GSK3β are expressed in the SCN and are rhythmically phosphorylated, peaking in the late night to early morning [278,279]. Lithium administration augmented phosphorylation of GSK3 and reduced total levels of GSK3α in a time-of-day-dependent manner [277]. Subsequently, Iitaka et al. (2005) demonstrated that inhibition of GSK3β in NIH-3T3 cells by lithium treatment induced GSK3β phosphorylation and delayed the phase of clock gene oscillations, whereas overexpression of GSK3β triggered a phase advance [278]. Iitaka et al. (2005) further showed that GSK3β physically associated with PER2 in cultured cells and murine brains, and phosphorylated PER2 in vitro [278]. Importantly, silencing of GSK3β inhibited the nuclear translocation of PER2, providing a mechanism for the period-lengthening effects of lithium [278]. Strangely, chronic treatment of SCN slices with the GSK3 inhibitor, CHIR-99021, shortened the period and increased the amplitude of PER2::LUC bioluminescence rhythms ex vivo, suggesting that GSK3 may participate in a feedback mechanism to regulate the molecular clock [279]. 

However, other studies have shown that other clock proteins may also be targeted by GSK3β. Harada et al. (2005) demonstrated using cultured cells that GSK3β can phosphorylate mCRY2 at Ser-553 and promote its degradation via the proteasomal pathway [280]. Phosphorylation of mCRY2 at Ser-557, which primes GSK3β-mediated phosphorylation at Ser-553, was found to occur in the SCN, suggesting a role of GSK3β-dependent mCRY2 degradation in central timekeeping [281]. GSK3β was also shown to phosphorylate Rev-Erbα at Ser55 and Ser59, enhancing its stability in cultured cells [282]. Whether this mechanism operates within the SCN is unknown. Furthermore, GSK3β can bind to and phosphorylate CLOCK, triggering its degradation by inducing phosphorylation within a conserved phospho-degron site [283]. 

Several mouse models have been used to examine the role of GSK3 in circadian rhythms. Using knock-in mice that expressed constitutively active forms of GSK3α and GSK3β, Paul et al. (2012) showed that constitutive GSK3 activity lengthened the period, reduced the amplitude of behavioral rhythms, and resulted in more fragmented activity patterns [284]. These effects were only observed in double knock-in mice, not single mutant animals [284]. Double knock-in mice also showed disrupted SCN firing patterns, with heightened firing frequencies in the subjective night compared to controls [284]. Similarly, Lavoie et al. (2013) found that mice that were haploinsufficient for GSK3β (GSK3β^+/-^) had lengthened behavioral period [285]. Paul et al. (2016) subsequently demonstrated that CHIR-99021 could suppress action potential firing of SCN neurons through a decrease of a persistent sodium current in the subjective day [286]. 

## 4. Neuromodulation in the *Drosophila* Pacemaker

### 4.1. Neuropeptides

#### 4.1.1. Pigment Dispersing Factor (PDF)

PDF is the most extensively studied neuropeptide within the fly clock network, and is exclusively expressed in the l-LN_v_s and s-LN_v_s [29,287]. The receptor for PDF is orthologous to vertebrate VPAC2. Thus, the roles of PDF-PDFR and VIP-VPAC2 signaling within the circadian timekeeping system of flies and mammals are broadly conserved. Under LD conditions, genetic ablation of *Pdf* resulted in a complete loss of morning anticipation and an advance in evening anticipatory behavior [29]. Upon release into DD, *Pdf^01^* (null) flies showed a gradual loss of rhythmicity whereby the majority of flies retained rhythmic behavior during the first two cycles only to become arrhythmic by the third cycle [29]. The small fraction of rhythmic flies exhibited weak, short free-running rhythms of approximately 23 h [29]. Consistently, endogenous oscillations of *tim* and *cry* transcripts in the clock neurons of *Pdf^01^* flies were normal on the first day of DD (DD1); however, by DD4, the amplitude of *tim* rhythms was significantly attenuated in all DN, LN_v_, and LN_d_ clock neurons [30]. PER protein rhythms persisted under DD, but became progressively weaker in s-LN_v_s and LN_d_s [31]. In addition, synchrony in PER subcellular distribution was markedly perturbed [31,32]. Whereas PER protein in the LN_v_ and LN_d_ neurons of wild-type flies was predominantly cytoplasmic at circadian time (CT) 17 and exclusively nuclear during the rest of the circadian cycle, nuclear-to-cytoplasmic shuttling of PER was observed at variable phases spanning CT 11 to CT 19 in LN_v_ and LN_d_ neurons of *Pdf^01^* mutants [31]. PDF is reported to have differential effects on clock oscillations within the circadian network [32]. PDF enhances synchrony in *per* cycling in s-LN_v_ and a subset of DN_1_ (i.e., DN_1a_) neurons [32]. In contrast, in *Pdf^01^* flies, CRY-positive DN_1p_ neurons immediately lose PER cycling after transfer into DD, suggesting a direct role for PDF in driving molecular rhythms in this subset of cells [32]. PDF lengthens the period of molecular oscillations in s-LN_v_s, the PDF-negative fifth s-LN_v_, a subset of DN_1_ neurons, and CRY-positive LN_d_s, an effect that is consistent with the short-period phenotype of *Pdf^01^* mutants and the ability of PDF to effectively stabilize PER and TIM proteins (Figure 2B) [32,288,289]. Conversely, enhanced PDF expression was associated with short period oscillations in CRY-negative LN_d_s, but had no effect on the generation of synchronous, high-amplitude rhythms in CRY-negative LN_d_, the fifth s-LN_v_, DN_1p_, DN_2_, and DN_3_ neurons [32]. 

Similar to VIP, PDF in *Drosophila* enables different populations of pacemaker neurons to act in a unified manner, maintaining coherence within the pacemaker network. This is possible because PDF-expressing l-LN_v_s and s-LN_v_s extend projections to the contralateral LN_v_s through the posterior optic tract or to the dorsal clock neurons in the dorsal protocerebrum, respectively [29,290]. The release of PDF from the s-LN_v_ projections terminating at the dorsal protocerebrum has been shown to be rhythmic [290]. Interestingly, King et al. (2017) identified a peptidergic circuit that controlled rhythmic locomotor activity in *Drosophila* [291]. PDF stimulation of DN_1_s was shown to activate circadian output neurons in the pars intercerebralis (PI), which signal via the neuropeptide diuretic hormone 44 (DH44) to neurons in the subesophageal zone (SEZ) [291]. SEZ neurons, in turn, were shown to communicate to the ventral nerve cord via the neuropeptide hugin, an ortholog of mammalian neuromedin U [291]. 

The G-protein coupled PDF receptor (also referred to as “Han” or “Groom of PDF”) is widely expressed in diverse neuronal groups including some clock neurons: namely, a subset of the DN_1_s and DN_3_s [292,293,294]. There are reports of additional PDFR-expressing clock neurons including some or all of the l-LN_v_s, and some LN_d_s and s-LN_v_s [292,295]. The circadian behavior of *pdfr* mutants phenocopied that of *Pdf^01^* flies, and was rescued by the restricted overexpression of the receptor in all *per*-expressing clock neurons [292,293,294]. Similar to *Pdf^01^* flies, PER oscillations in *pdfr* mutants showed high variation in the phase of PER nuclear localization in s-LN_v_s and an advanced peak expression in two clusters of clock neurons, the s-LN_v_s and LN_d_s [293]. Similar to mammalian VPAC2, PDFR signals predominantly through cAMP and weakly through Ca^2+^ as assayed in S2 and HEK293 cells [292,294]. In vivo, real-time detection of cAMP in PDF-expressing clock neurons revealed that only s-LN_v_s responded to PDF with a robust increase in cAMP [296]. On the other hand, l-LN_v_s showed infrequent and extremely weak cAMP induction in response to PDF stimulation, consistent with the low or absent expression of PDFR in these neurons [296]. Notably, ectopic expression of PDFR in the l-LN_v_s conferred robust PDF-induced activation of cAMP, whereas overexpression in s-LN_v_s amplified their response to PDF [296]. Other non-PDF expressing clock neurons, including DN_1_s, DN_2_s, DN_3_s, LN_d_s, and the PDF-negative fifth s-LN_v_, also showed a pronounced increase in cAMP upon PDF stimulation [296]. 

*Drosophila* clock neurons show circadian-gated sensitivity to PDF, an effect dependent on PDFR signaling and mediated by the small Rho-related G protein, RalA [297]. *In vivo*, s-LN_v_s show a peak in PDF-mediated cAMP induction during the early morning hours (ZT/CT1-4) that is 50% higher than the rest of the circadian cycle [297]. This effect persists even when PDFR is ectopically overexpressed in *pdfr* mutant flies, indicating that it is independent of the rhythmic *pdfr* expression in s-LN_v_s [297]. Rather, these effects are dependent on RalA, as expression of a constitutively active or a dominant-negative form of this small GTPase abolished the oscillations in PDF sensitivity of s-LN_v_s [297]. 

PDFR signaling also acts to modulate the phase of Ca^2+^ rhythms in the different clusters of clock neurons. Although the *Drosophila* pacemaker network displays synchrony in other aspects, Ca^2+^ rhythms between different neuronal clusters are asynchronous and coincide with their role in behavior [298]. *In vivo*, the s-LN_v_s drive morning activity and consequently show peak Ca^2+^ levels preceding dawn, whereas the LN_d_s and the PDF-negative fifth s-LN_v_ influence evening activity and show peak Ca^2+^ levels before dusk [298]. Mutations in *pdfr* delayed the phase of Ca^2+^ rhythms in LN_d_ and DN_3_ neurons such that peak levels shifted from CT8 and CT16, respectively, to CT0, roughly in synchrony with Ca^2+^ oscillations in M cells [298,299]. Interestingly, Ca^2+^ oscillations in M cells were not affected by the absence of PDFR signaling [298]. 

The variable effects of PDF signaling on molecular oscillations, Ca^2+^ rhythms, and cAMP induction in the different clock clusters may be explained by the mosaic expression of PDFR. However, an alternative explanation stems from the finding that PDFRs in different clusters of clock neurons signal via distinct isoforms of adenylyl cyclases (ACs), which may differ in their enzymatic properties and mode of regulation [300,301]. For instance, in s-LN_v_s, PDFR signals through AC3, whereas in LN_d_s it utilizes AC78C and at least one other unidentified adenylyl cyclase [300,301]. Thus, PDF-induced PDFR signaling activates Gαs (also known as Gsα60A) and specific AC isoforms, ultimately leading to cAMP production. cAMP can then activate various downstream signaling cascades including PKA, which targets components of the endogenous clock such as PER and TIM and channels the effects of PDF-PDFR signaling to the core clockwork (Figure 2B) [288,289,296,300,301,302].

#### 4.1.2. Neuropeptide F (NPF) and Small NPF (sNPF)

The neuropeptide Y(NPY)-like peptides, Neuropeptide F (NPF) and short Neuropeptide F (sNPF), and their respective cognate receptors, NPFR1 and sNPFR, are widely expressed in the *Drosophila* central nervous system (CNS), and play diverse roles in various neuronal circuits including sleep and circadian pacemaking [303,304,305,306,307]. Both NPF and sNPF have been detected in *Drosophila* pacemaker neurons, and are clock-regulated in a manner that is consistent with a role in regulating locomotor activity under LD conditions [308,309,310,311]. This contrasts with mammalian NPY, which is not expressed in central clock neurons of the SCN, but in intergeniculate leaflet neurons that project to the SCN to transmit photic and nonphotic information [312,313,314]. 

Of the six LN_d_s in the adult fly clock, two of the CRY-positive LN_d_s express sNPF in both male and female flies, and three other LN_d_s in males but fewer in females express NPF, indicating that the expression of NPF is sexually dimorphic [308,315,316]. NPF expression was also seen in the PDF-negative fifth s-LN_v_ and a subset of the l-LN_v_ and possibly s-LN_v_ neurons [309,316]. sNPF is expressed in four and two of the PDF-positive s-LN_v_s in the adult and larval clocks, respectively, but is absent in the PDF-negative fifth s-LN_v_ and the l-LN_v_s [315]. The two sNPF-expressing LN_d_s also express choline acetyltransferase, signifying potential regulation via cholinergic signaling (Figure 2A) [315]. With respect to the receptors, NPFR1 is expressed in a subset of DN_1_ and LN_d_ clock neurons, whereas sNPFR is found in the l-LN_v_s [309,310]. Transcriptome profiling of specific neuronal populations using RNA-sequencing demonstrated that the mRNA expression of NPF, sNPF and their cognate receptors may extend to other clock neurons than those indicated above; however, protein expression at these additional sites remains to be examined [311].

NPF expression in LN_d_s requires a functional circadian clock, as indicated by the absence of *npf* mRNA expression in the clock mutants, *Clk^Jrk^* and *cyc^02^* [308]. In the adult brain, *npf* and *npfr* transcripts exhibit rhythmic expression with a peak at ZT22.5 and ZT4.5–7.5, respectively, under LD conditions [309]. At the protein level, NPF abundance in the LN_d_s and l-LN_v_s peaks at ZT8; however, no rhythms in NPFR1 were detected in adult brains [309]. Ablation of *npf*-expressing clock neurons in male flies results in an advanced peak of evening anticipation, suppression of late evening activity, and reduced evening peak amplitude [308,316]. However, inconsistent effects were observed when *npf*-expressing cells were ablated in female flies [308,316]. The effect on evening activity is primarily a consequence of the loss of the *Pdf*-negative subset of *npf*-expressing clock neurons [316]. Ablation of *Pdf*-positive neurons alone results in advanced evening anticipation with no change in amplitude; however, ablating *npf*-expressing clock neurons in conjunction with *Pdf*-positive cells appears to have an additive effect, leading to a potentiated advance in evening activity and a marked reduction in activity amplitude [316]. Under free-running conditions, flies in which *npf*-positive clock neurons have been ablated exhibit a longer period but no pronounced change in rhythmicity [316]. Importantly, the observed period lengthening in these animals is independent of loss of NPF signaling per se, since knocking down the expression of *npf* or *npfr1*, without eliminating the cells themselves, did not alter free-running rhythms [309]. On the other hand, evening anticipatory behavior was impacted by *npf* or *npfr1* gene silencing [309]. In addition to circadian rhythms, NPF has also been shown to regulate fly sleep behavior. He et al. (2013) reported the sleep-promoting effects of NPF-NPFR1 signaling, whereas Chung et al. (2017) demonstrated that wakefulness was instead promoted by activation of NPF-NPFR1 signaling in *cry*-positive, *npf*-expressing clock neurons [317,318]. The reason behind this discrepancy remains unknown.

In the context of circadian timing, sNPF released from the M and E cells serves to modulate the phase of Ca^2+^ activation in the DN_1_ neuronal cluster via daytime suppression of Ca^2+^ levels [299]. Accordingly, knockdown of *snpf* in the M cells enhanced Ca^2+^ accumulation at dawn in DN_1_s, abolished LD morning anticipation, and delayed the phase of the M peak under free-running conditions [299]. Restricting *snpf* knockdown to the E cells did not impact DN_1_ Ca^2+^ rhythms or locomotor activity, suggesting that sNPF from M cells is capable of supporting rhythmic DN_1_ activity in the absence of E cell-derived sNPF [299]. However, E cell-derived sNPF can play a more prominent role in the daytime suppression of DN_1_ Ca^2+^ activity but only in the absence of input from the s-LN_v_s [299].

In contrast to the excitatory and cAMP-activating effects of PDF-PDFR signaling, activation of sNPF-sNPFR signaling reduced membrane excitability and cAMP levels via Gα_0-_dependent signaling, at least in larval motor neurons [319]. These effects mirror those of mammalian NPY, which has been shown to suppress excitability of SCN neurons [320] and reduce cAMP accumulation in a pertussis toxin-sensitive manner [321]. The inhibitory effect of sNPF on neuronal firing may be crucial for its role as a sleep-promoting peptide, given that sNPF secreted from s-LN_v_s has been shown to suppress dopamine-dependent increase in cAMP in the wake-promoting, sNPFR-positive l-LN_v_s [310,319,322]. It would therefore appear that, within the clock network, sNPF acts as an inhibitory neuromodulator to regulate circuit-controlled behaviors such as sleep. However, in a conflicting report, sNPF-deficient flies were found to show enhanced sleep via a mechanism that may involve sNPF-mediated activation of cAMP, as demonstrated in the BG2-c6 neural cell line, which was established from the CNS of *Drosophila* third instar larvae [323]. These contradictory results suggest that the downstream effects of sNPF signaling may be highly cell type-specific or context-dependent, as is the case for PDF [300,301]. 

#### 4.1.3. Ion Transport Peptide (ITP)

The ion transport peptide (ITP) is an insect neuropeptide that is expressed in one of the CRY-positive, NPF-positive LN_d_s as well as the PDF-negative fifth s-LN_v_ (Figure 2A) [315]. ITP is released from these cells into the dorsal protocerebrum through projections that terminate in the PI in a clock-controlled and rhythmic manner under both LD and DD conditions [324]. Under LD, flies in which *itp* was knocked down in *tim*-expressing cells showed normal phasing of M and E activity bouts [324]. However, relative activity levels were perturbed in these flies, such that there was hyperactivity during the night, reduced daytime activity, and lower evening:morning activity counts [324]. Under DD conditions, *itp* knockdown flies showed normal rhythmic activity with a modest but significant increase in period [324]. In contrast, when overexpressed in *tim*-positive cells, ITP induced aberrant LD behavior characterized by loss of M and E activity bouts and a slight reduction in PER amplitude in s-LN_v_ and LN_d_ neurons [324]. Under DD, these flies exhibited behavioral arrhythmia, which could not be fully explained by the damping of PER cycling in the s-LN_v_s [324]. A more likely explanation for the arrhythmic behavior evoked by ITP overexpression may be the observed constant release of both ITP and PDF into the dorsal brain, affecting clock output regions, including the PI, that are known to control rhythmic locomotor activity [40,291,324]. The findings that ITP affects rhythmic behavior, PER cycling, and rhythmic PDF release suggest a pivotal role for this insect neuropeptide in circadian pacemaking. However, identifying and determining the expression pattern and distribution of the ITP receptor in the fly brain will be critical in understanding the neural basis for ITP-mediated regulation of the fly clock [324]. 

#### 4.1.4. Diuretic Hormone 31 (DH31)

Within the *Drosophila* clock network, the mRNA expression of diuretic hormone 31 (DH31) has been mapped to the LN_v_s and the DN_1_s, including the DN_1a_ and DN_1p_ subset of dorsal neurons [311,325,326,327]. In these neurons, the abundance of DH31 transcripts oscillates, reaching peak levels during the morning phase [311]. DH31 expression within the fly clock network has been implicated in the circadian control of sleep regulation, temperature preference rhythm (TPR), and circadian locomotor activity [325,326,327]. As a wake-promoting peptide, DH31 is released from PDFR-positive DN_1_s in response to PDF stimulation by s-LN_v_s, resulting in sleep suppression in anticipation of dawn [325]. DH31-mediated activation of PDFR in DN_2_ neurons modulates TPR, promoting the preferred temperature decrease that occurs at the day-to-night transition [326]. Interestingly, calcitonin receptor, the mouse homolog of the DH31 receptor, is expressed in the dorsal SCN and was recently shown to mediate body temperature fluctuations during the animal’s active phase [328], suggesting that the functions of these receptors are conserved between flies and mammals. In addition, free-running locomotor activity rhythms appear to be co-modulated by DH31 and PDF: a loss-of-function mutation in the *Dh31* locus on its own had no impact on rhythmic behavior but evoked severe behavioral arrhythmia when combined with the *Pdf^01^* mutation [327]. Notably, the arrhythmic phenotype was stronger in the double mutants than the *Pdf^01^* single mutants [327], and rhythmicity was restored to a level comparable to the *Pdf^01^* mutants upon overexpression of DH31 [327]. On the other hand, overexpression of PDF resulted in full restoration of rhythmic behavior in the double mutants [327]. Using tethered ligands, it was deduced that PDF and DH31 acted in a hierarchical fashion on DN_1p_ neurons to regulate free-running rhythms, with PDF serving as the primary regulator and DH31 assuming a subordinate function [327]. The downstream mechanisms of DH31 signaling remain to be identified.

#### 4.1.5. CChamide 1

*Drosophila* CChamide 1 (CCHa1) and CChamide 2 (CCHa2) were recently identified in a screen for the endogenous ligands of the GPCR, bombesin receptor subtype 3 (BRS3) [329,330]. In particular, CCHa1 is expressed in a rhythmic manner (under LD and DD) in the cell bodies and terminals of DN_1a_ clock neurons projecting to the s-LN_v_s (Figure 2A) [331]. CCHa1 receptor (CCHa1R), the fly homolog of the mammalian GRP receptor, bombesin 2 (BB_2_), is expressed in the l-LN_v_ and s-LN_v_ neuronal clusters, with mRNA levels reaching a peak during the late night to early morning [311,331]. Within the clock network, CCHa1 regulates the phase and amount of morning activity under LD and DD conditions, but has no role in maintaining robust free-running rhythms [331]. Consistent with its role in morning activity, CCHa1 affects the phase of molecular clock oscillations and elicits cAMP accumulation via CCHa1-CCHa1R signaling in the s-LN_v_s [331]. However, the impact of CCHa1 knockdown on molecular oscillations was not restricted to the M cells, but was observed in the majority of clock neurons, suggesting a broad function of CCHa1 signaling within the clock network [331]. Interestingly, CCHa1 knockdown flies did not exhibit the same degree of temporal change in the axonal arborizations and PDF levels of s-LN_v_ terminals in the dorsal protocerebrum compared to control flies [331]. Specifically, CCHa1 knockdown led to a more extensive pattern of axonal arborization and a significant increase in PDF immunoreactivity in these projections in the late day [331], suggesting that the widespread effects of CCHa1 knockdown on the clock network may be through altered PDF signaling to other clock cells within the network. 

#### 4.1.6. Allatostatin-C (AstC)

Allostatin-C (AstC) is another clock-controlled neuropeptide that was first detected in the *Drosophila* pacemaker network via an RNA-sequencing screen of individual neuronal clusters and later confirmed to be highly expressed within the DN_1p_, DN_3_ and LPN neuronal groups (Figure 2A) [41,311]. The AstC receptor 2 (AstC-R2), the fly homolog of the mammalian somatostatin-galanin-opioid receptor family, is expressed in the LN_d_ clock neurons [311,332]. Within the DN_1p_ cluster, AstC expression oscillates in a time-of-day-dependent manner, reaching peak and trough expression during the late night (ZT20) and early morning (ZT0-4), respectively [41]. Knocking down either AstC or AstC-R2 expression in clock neurons resulted in a delay in the phase of evening activity peak under short and long photoperiods [41]. Ex vivo calcium imaging revealed that application of AstC directly inhibits a single neuron within the LN_d_ cluster of E cells, potentially accounting for the perturbed phase of the evening peak when AstC signaling is disrupted [41]. Interestingly, somatostatin (SST) has been implicated in mammalian photoperiodic adaptation, suggesting a conserved function with *Drosophila* AstC-AstC-R2 [333,334,335]. Moreover, the rhythmic expression of SST has been described in the rat SCN [336,337]. Notably, the functional significance of SST in circadian timekeeping has only been explored within the context of phase resetting of behavior and electrical activity of the SCN [338,339]. It remains to be determined whether SCN-resident SST neurons serve a function in photoperiodism. 

#### 4.1.7. IPNamide

Shafer et al. (2006) detected IPNamide immunoreactivity specifically in DN_1a_ neurons [340]. Given its localization, IPNamide, a product of the neuropeptide-like precursor 1 gene [341,342], may be another neuropeptide that potentially contributes to the regulation of the *Drosophila* clock circuit, although a specific function in circadian timekeeping has yet to be shown. 

### 4.2. Neurotransmitters

#### 4.2.1. Glutamate

There is ample evidence demonstrating the role of glutamatergic signaling in establishing circadian rhythmicity in *Drosophila*. Circadian behavior has been investigated in the context of the minimal larval clock circuit, which generates circadian rhythms in larval light avoidance. DN_1_ neurons in the larval brain as well as DN_1_ and DN_3_ neurons in the adult brain express vesicular glutamate transporter, indicating that these neurons are glutamatergic (Figure 2A) [343]. As they communicate with the s-LN_v_s, it is not surprising that larval and adult LN_v_ neurons express the *Drosophila* metabotropic glutamate receptor (DmGluRA) [343]. Glutamate application attenuated intracellular Ca^2+^ levels in the larval s-LN_v_s [343]. Interfering with DmGluRA expression in the larval or adult brain resulted in perturbations in photophobic behavior and locomotor activity patterns, respectively [343]. Overall, these observations indicate that dorsal neuron-dependent glutamatergic signaling modulates circadian behavior by acting on DmGluRA expressed by the LN_v_s [343]. A separate study demonstrated that neuronal excitability of larval LN_v_s and larval DN_1_s peaked in antiphase to one another, such that low CLK/CYC activity during dawn enhanced the excitability of LN_v_s but suppressed it in DN_1_s [344]. Larval DN_1_s were shown to suppress LN_v_ neuronal activity via inhibitory glutamatergic inputs that acted on glutamate-gated chloride channels expressed by LN_v_s [344]. This relationship between DN_1_s and LN_v_s is necessary not only for the rhythmic patterns in light avoidance observed in larvae, but also in the generation of robust locomotor rhythms in adult flies [344]. Lastly, glutamate has also been implicated as a non-canonical synchronizing signal that is released from DNs to modulate LN_v_s via the DmGluRA [345]. Collins et al. (2014) demonstrated that PDF and glutamate were released in antiphase to each other, activating PDF receptors and mGluRA at different times of day to drive cAMP oscillations within larval LN_v_s [345]. The cooperative actions of PDFR and DmGluRA signaling were important for driving robust TIM oscillations and maintaining molecular synchrony in larval LN_v_s and adult s-LN_v_s [345].

#### 4.2.2. GABA

GABAergic modulation of the central pacemaker exhibits a high degree of conservation between flies and mice. *Drosophila* express three GABA_B_R genes, GABA_B_-R1, -R2, and -R3 [346,347]. Expression of GABA_B_-R2 and -R3 have been observed in the LN_v_s of both adult and larval brains [346,347]. GABA has been shown to act on LN_v_s, functioning as a slow-acting inhibitory neurotransmitter [346,348]. Application of GABA to dissociated larval s-LN_v_s or intact adult s-LN_v_s attenuated intracellular Ca^2+^ release via activation of metabotropic GABA_B_R, resulting in a blockade of spontaneous Ca^2+^ oscillations [346,348]. Genetic ablation of GABA_B_-R3 in LN_v_s increased the period of locomotor activity rhythms in adult flies, whereas hyperexciting GABAergic neurons induced behavioral arrhythmia, which coincided with perturbations in the cyclic expression of the clock genes *period* and *vrille* [347]. 

At the molecular level, GABA_B_-R3 signaling in the LN_v_s appears to involve the parallel activation of both G_s_ and G_o_ signal transduction pathways, which have additive effects on period length in adult flies [347,348]. Interestingly, GABA_B_-R2 does not appear to be critical for maintaining circadian rhythmicity, as genetic manipulations that downregulated GABA_B_-R2 expression did not alter adult locomotor activity rhythms [349]. Lastly, it was demonstrated that the light-input factor Quasimodo (Qsm) mediated acute and daily light effects driving the rhythmic excitability of the adult l-LN_v_s [350]. The daily variation in neuronal activity of l-LN_v_s was dependent on the expression level of Qsm, as its overexpression elicited a constitutive, less active “night” state, and its knockdown produced a more active “day” state [350]. These effects were shown to be mediated by the Shaw K^+^ channel (dK_v_3.1) and the Na^+^, K^+^, Cl^−^ cotransporter (NKCC), the latter of which physically interacts with Qsm and can switch the effects of GABA from inhibitory to excitatory [350].

GABAergic signaling has also been implicated in the rhythmic coupling of the circadian clock to sleep/wake cycles. GABA_A_Rs in adult l-LN_v_s are expressed rhythmically and undergo rhythmic degradation [351]. The E3 ligase, F-box and leucine rich repeat protein 4 (*Fbxl4*), a CLK-dependent gene, promotes the ubiquitination and turnover of GABA_A_Rs [351]. Knockdown of *Fbxl4* expression attenuated sleep onset latency and increased sleep duration by modulating rhythmic GABA sensitivity, indicating that *Fbxl4* is an output molecule that links the central clock to the regulation of sleep [351]. Similarly, the *Drosophila* mutant, WIDE AWAKE, exhibited deficits in sleep onset [352]. WAKE, the gene that is affected in WIDE AWAKE mutants, is expressed cyclically in a CLK-dependent manner in LN_v_s and was shown to regulate the expression of the GABA_A_R, Resistant to dieldrin (*Rdl*), which has well-established roles in sleep latency [33,35,352]. Notably, both *Fbxl4* and WIDE AWAKE mutants exhibited normal locomotor rhythms, indicating that these clock-output molecules act specifically to modulate sleep [351,352].

#### 4.2.3. Serotonin

Serotonin immunoreactivity has been observed in neuronal processes near the dendrites and the dorsal terminals of s-LN_v_s in both adult and larval flies [353]. Levels of serotonin decline under DD conditions in the adult fly brain, suggesting that serotonergic modulation of the clock is dependent on external photic cues [354]. Application of serotonin to larval LN_v_s elicited a substantial reduction in intracellular Ca^2+^ levels [353]. Larval and adult LN_v_s express d5-HT1B, an ortholog of the mammalian 5-HT1A receptor; genetic perturbations of d5-HT1B altered behavioral and molecular responses to light in adult flies [354]. Overexpression of d5-HT1B attenuated light-induced phase shifts, which coincided with increased expression of TIM [354]. In contrast, knockdown of d5-HT1B expression hypersensitized the adult flies to the effects of light [354]. At the molecular level, d5-HT1B-dependent serotonergic signaling reduced the activity of the GSK3 ortholog, SHAGGY, which mediates phosphorylation-dependent destabilization of TIM [354]. Interestingly, the 5-HT2 agonist, 1-[2,5-dimethoxy-4-iodophenyl]-2-aminopropane (DOI), has been shown to increase morning locomotor activity and eliminate anticipatory behavior in adult flies, effects that were attenuated in the absence of the 5-HT2 receptor, 5-HT(2)Dro [355].

#### 4.2.4. Acetylcholine

Cholinergic signaling is integral to the maintenance of circadian rhythms in *Drosophila*. LN_v_s in the adult and larval brain express excitatory nicotinic ACh receptors, including the nAChR subunits Da2, ALS, and ARD [356,357]. Bath application of nicotinic agonists induced Ca^2+^-dependent excitation of both s-LN_v_s and l-LN_v_s in the adult brain [348,357]. Furthermore, electrophysiological studies using the nicotinic ACh receptor antagonist, α-bungarotoxin, in adult flies have demonstrated that action potential-dependent (or tetrodotoxin-sensitive) nAChR synaptic signaling is necessary for rhythmic l-LN_v_ activity [356]. Larval clock entrainment and larval light avoidance are mediated by distinct cholinergic pathways that involve ACh release from the blue-sensitive rhodopsin5 (rh5) and green-sensitive rhodopsin6 (rh6) photoreceptors, respectively, both of which innervate the LN_v_s [358]. Lastly, it has been suggested that cholinergic signaling may serve as an input signal from the optic neuropil of the larval Bolwig organ to the dendrites of the LN_v_s to provide photic modulation of clock neurons in a manner that is Ca^2+^-dependent [357]. Notably, the H-B eyelets, which are the adult remnants of the Bolwig organ, utilize acetylcholine and histamine to convey photic information to clock neurons [25,359]. ACh release from the H-B eyelets induces Ca^2+^- and cAMP-mediated activation of the M cells, promoting morning activity and mediating adaptation to high light intensities, whereas histamine release from the H-B eyelets inhibits the activity of the l-LN_v_s [25,53]. Interestingly, this inhibition of the l-LN_v_s potentially ceases under long photoperiods, when light-evoked excitation of the l-LN_v_s may be mediated directly by CRY or indirectly through the release of ACh from the compound eyes [25,360,361]. The activated l-LN_v_s then modulate the timing of the E activity peak by direct neurotransmission to the E cells in the accessory medulla, a neuropil where projections from various clock neurons and visual structures converge to mediate photic entrainment [25,52]. 

#### 4.2.5. Glycine

In the adult *Drosophila* brain, the inhibitory neurotransmitter glycine is expressed in the LN_v_s, where it acts to organize rhythmicity of the circadian network (Figure 2A) [362]. Bath application of glycine reduced the firing frequency of DN_1p_ neurons, the targets of glycinergic s-LN_v_s, in isolated fly brains [362]. Attenuating glycine neurotransmission from *Pdf*-expressing cells via knockdown of the glycine transporter, CG5549, or the serine hydroxymethyltransferase, Shmt, the latter of which is required for glycine synthesis, resulted in a lengthening of the behavioral period [362]. Furthermore, disrupting glycinergic transmission through *CG5549* knockdown reduced the stability of the circadian network, such that its effects mimicked the clock instability that is commonly observed under dim constant light [362]. The actions of glycine are mediated in part by the GlyR subunits, CG12344, CG7589, and Grd, since RNAi-mediated knockdown of these GlyR subunits perturbed circadian rhythmicity by altering the glycinergic inhibitory tone of the network [362]. 

## 5. Protein Kinases Implicated in Circadian Timekeeping in *Drosophila*

Numerous protein kinases have been demonstrated to regulate the *Drosophila* circadian timekeeping system (Figure 2B). In most cases, the studies have focused on the potential kinase-substrate relationship between a particular protein kinase and a core clock protein, and the effects of phosphorylation by that kinase on the properties of the clock protein. Phosphorylation has been observed to alter the stability, nucleocytoplasmic trafficking, and the activity of a clock protein. As a result, most of the kinases described below affect the period or stability of the molecular clockwork when their expression or activity is manipulated. In contrast to our knowledge of protein kinases pertinent to the mammalian timekeeping system, much less is known about the upstream signaling events that might impinge on the *Drosophila* kinases, or the roles that these kinases may play in the entrainment process. One can clearly envision the importance of protein kinase signaling in entrainment, especially in the case of CRY-independent photoreception, where photoreceptive organs must transmit the light information to clock neurons via a synaptic mechanism that involves receptor or channel activation. Intracellular signaling events must then be engaged to couple the photic signal to the molecular clockwork.

### 5.1. Doubletime (DBT)

Price et al. (1998) isolated two semi-dominant mutations, termed doubletime-short (*dbt^S^*) and doubletime-long (*dbt^L^*), that respectively shortened or lengthened the behavioral period [363]. The mutations resided within the *doubletime* gene, which encodes a protein that is structurally homologous to mammalian CKIε and CKIδ [364]. Despite exerting opposite effects on circadian period, both the *dbt^S^* and *dbt^L^* mutations, when introduced into the orthologous CK1 protein in *Xenopus*, resulted in a reduction in kinase activity, at least towards its canonical substrate, casein [365]. Other mutations in *dbt* have also been identified that affect period length and circadian rhythmicity, including two mutants, *dbt(ar*) and *dbt(EY02910*), that rendered flies arrhythmic, and another, *dbt^P^*, that abolished the expression of *dbt* along with cycling of *per* and *tim* transcript in the fly larvae [363,364,366,367,368,369]. *dbt^P^* mutants exhibited constitutively elevated levels of PER in a hypophosphorylated form, which, along with the observed physical association between DBT and PER, suggested that PER may be a target of DBT-mediated phosphorylation [363,364]. Likewise, the *dbt(ar*) adult flies had constitutively high levels of PER which accompanied the behavioral arrhythmia [366]. The *dbt(EY02910*) mutants displayed decoupled PER and TIM oscillations in the LN_v_s, suggesting a potential role of DBT in clock synchrony [369]. Importantly, the per-short (*per^S^*) mutation, a substitution at Ser589 that shortens the circadian period, rescued the phenotype of *dbt(ar*) mutants, restoring rhythms in behavior and PER abundance [366,370]. Rothenfluh et al. (2000) hypothesized that the mutations in *dbt* and *per^S^* affected the same molecular event that controlled the turnover of PER, a speculation that proved to be correct when Chiu et al. (2008) [371] demonstrated that DBT is the kinase for Ser589 [366]. In addition to Ser589, DBT was also shown to phosphorylate PER at Ser47: phosphorylation at Ser47 and neighboring residues creates a high-affinity binding site for the F-box protein SLIMB [372]. Ko et al. (2002) had previously demonstrated that DBT triggered hyperphosphorylation of PER and its degradation by the proteasome [373]. 

In addition to affecting PER stability, DBT has been shown to control the timing of nuclear entry of PER. The *dbt^S^* mutation resulted in a delayed nuclear accumulation of PER protein (specifically in photoreceptor cells), and delayed the upswing but accelerated the downswing in *per* transcripts in whole head extracts [374]. However, Bao et al. (2001) did not observe a change in total PER abundance in *dbt^S^* mutants [374]. Cyran et al. (2005) observed that PER was constitutively nuclear in the s-LN_v_s in a *tim*-null background, but only when DBT kinase activity was also inhibited [375]. Cyran et al. (2005) hypothesized that DBT-mediated phosphorylation promoted cytoplasmic retention of PER [375]. They further suggested that the findings of Bao et al. (2001) may, in fact, be due to enhanced, rather than reduced, DBT kinase activity directed against PER in *dbt^S^* mutant flies [374,375]. Kivimäe et al. (2008) identified several putative DBT phosphorylation sites on PER and showed that phospho-occupancy of specific residues affected the repressive effects of PER on CLK-mediated transcription [376]. 

Lastly, Kloss et al. (2001) showed that the expression of endogenous (wild-type) DBT in whole head extracts does not exhibit circadian variation [377]. On the other hand, the nucleo-cytoplasmic distribution of DBT in lateral neurons is phase-dependent and roughly matches that of PER/TIM, localizing primarily to the nucleus in the late night/early day and to the cytoplasm in the first half of the night [377]. DBT was shown to physically associate with PER and PER/TIM complexes throughout the circadian cycle, suggesting that DBT may influence PER activity or function in both subcellular compartments [377].

Besides PER, CLK has also been suggested to be a target of DBT. Kim and Edery (2006) showed the DBT is required for the hyperphosphorylation of CLK in fly heads and S2 cells [378]. DBT-dependent phosphorylation of CLK reduced its stability and its transcriptional activity, albeit modestly [378]. These findings were mirrored by Yu et al. (2009); however, they reported that the catalytic activity of DBT was not required for it to promote the phosphorylation of CLK or the repression of CLK’s transcriptional activity [379]. They speculated that DBT may be recruiting another kinase that directly phosphorylates CLK [379]. 

### 5.2. CK2

Konopka et al. (1991) identified a semi-dominant clock mutation, *andante*, in *Drosophila* that elicited a ~2 h lengthening of eclosion and activity rhythms [380]. Akten et al. (2003) later mapped the mutation to the CK2β gene [381]. The long behavioral period that was exhibited by *andante* flies and other mutants defective for CK2β could be rescued by expressing a wild-type CK2β transgene [381]. As expected, overexpression of CK2β on an otherwise wild-type background resulted in period shortening [381]. The *andante* mutation was predicted to interfere with CK2 activity by disrupting the dimerization of CK2β subunits and the assembly of the α2:β2 holoenzyme [381]. The expression of CK2 subunits do not appear to be rhythmic in the fly brain [381]. Importantly, CK2β expression is localized to the small and large LN_v_s, and the *andante* mutation resulted in a ~2 h delay in the nuclear accumulation of PER and TIM in the s-LN_v_s as well as an elevation in their steady-state levels [381].

Lin et al. (2002) identified a dominant mutant, *timekeeper (Tik*), that produced a period lengthening similar to the *andante* mutant [382]. The *Tik* mutation mapped to the CK2α gene and resulted in a disruption of CK2α catalytic activity [382]. The expression of CK2α was detected in PDF-expressing neurons in adult flies [382]. Restricting the expression of CK2α*^Tik^* to TIM- or PDF-positive cells, or conditionally inducing its expression in adult PDF neurons, lengthened behavioral period in a manner that was dependent on gene dosage [383]. Paradoxically, overexpression of wild-type CK2α in TIM- and PDF-expressing cells also elicited period lengthening [384]. Overexpressing CK2α*^Tik^* in PDF neurons delayed nuclear entry of PER by >4 h and elevated trough levels of PER at the transcript and protein level, suggesting that feedback repression is disrupted [383]. The effects on PER appear to be direct, as in vitro kinase assays revealed that PER is phosphorylated by CK2α, specifically at Ser151 and Ser153 [382,384]. Phospho-inactive PER(S151-153A) mutant flies displayed the expected period lengthening, as did, paradoxically, the phosphomimetic PER(S151-153D) mutant flies [382,384]. Overall, these data indicate that CK2 targets PER to control the timing of feedback repression and thus the pace of the circadian clock.

In addition to PER, other clock proteins may also be targets of CK2α. Meissner et al. (2008) reported that the effects of CK2α*^Tik^* on PER abundance were abolished on a *tim*-null background, and nuclear translocation of PER in *Tik*-expressing flies could be enhanced by light, which is known to trigger TIM degradation [385]. Furthermore, the long period of *Tik* mutants was partially rescued by the *tim(UL*) mutation, which may potentially interfere with CK2 phosphorylation [385]. These results suggest that TIM is mediating at least some of the effects of *Tik* on PER [385]. Overexpressing CK2α*^Tik^* resulted in cytoplasmic retention of TIM in the s-LN_v_s and elevated trough levels of TIM protein and mRNA [385]. More recently, Szabό et al. (2013) identified CLK as a direct target of CK2α [386]. Overexpression of CK2α*^Tik^* in *tim*-expressing cells induced the degradation of CLK, even on a *per*- and *tim*-null background [386]. Knocking down CK2β, on the other hand, had no effect on CLK stability [386]. Immunoprecipitation assays revealed a physical association between CK2α and CLK in the presence of PER in the fly head during the late night to early morning [386]. CK2α promotes the hyperphosphorylation of CLK, which stabilizes the protein but at the same time suppresses its transcriptional activity [386]. 

### 5.3. Shaggy/GSK3

Shaggy (SGG), the *Drosophila* ortholog of GSK3, was identified by Martinek et al. (2001) in a genetic screen for period-modifying genes [387]. Overexpression of SGG in *tim*-expressing clock cells resulted in a ~2–3-h period-shortening of locomotor rhythms [27,387]. Conversely, *sgg* hypomorphic mutants exhibited lengthened period [387]. In vitro assays revealed that mammalian GSK3β, which is 85% identical to SGG in the kinase domain, could phosphorylate *Drosophila* TIM [387]. Consistent with this, *sgg* hypomorphs had reduced levels of the hyperphosphorylated form of TIM in head extracts, and overexpression of *sgg* resulted in the premature nuclear entry of PER/TIM complexes in the lateral neurons of fly larvae [387]. 

Other studies confirmed the period-shortening effects of *sgg* overexpression in all PDF neurons or specifically s-LN_v_s [27,388]. Interestingly, *sgg* overexpression in PDF neurons resulted in period shortening, as indicated by an advanced clock phase on day 4 of DD, in s-LN_v_s but not l-LN_v_s [27]. In line with this, Top et al. (2016) found that overexpression of *sgg* in l-LN_v_s had no effect on behavioral period [388]. Top et al. (2016) provided additional mechanistic insights by showing that SGG-mediated phosphorylation of TIM consequently triggers a phosphorylation cascade mediated by CK2 [388]. In vitro kinase assays revealed that SGG phosphorylated TIM at Ser297/Thr301 [388]. Phosphomimetic mutations of these two residues promoted CK2-mediated phosphorylation at Thr305, Thr309, and Ser313 [388]. The onset of nuclear accumulation of PER/TIM complexes was delayed by mutating the SGG phosphorylation sites to alanine, suggesting that SGG-mediated phosphorylation regulates the onset of nuclear entry [388]. Similar phospho-deficient mutations of the CK2 sites of TIM did not alter the onset of PER/TIM nuclear entry; however, knockdown of CK2α increased the levels of PER/TIM that accumulated in the nucleus [388]. These effects on PER/TIM were observed in cell culture but also in vivo in s-LN_v_s, and were reflected in predictable changes in the behavioral period of flies bearing phosphomimetic or phospho-deficient mutations at the SGG or CK2 sites of TIM [388].

In addition to TIM, PER was shown to be a direct phosphorylation target of SGG [389]. Ser657 of PER is phosphorylated by SGG once PER is phosphorylated (and primed) at Ser661 by a proline-directed kinase [389]. The behavioral period of flies bearing a Ser-to-Ala substitution at residue 657 is increased by ~1 h, compared to ~2 h for flies bearing the same substitution at residue 661 [389]. PER(S657A) mutant flies likely experience delayed nuclear entry of PER in the s-LN_v_s, as empirically demonstrated in the PER(S661A) mutants [389].

### 5.4. NEMO/NLK

Two studies identified the proline-directed kinase, NEMO, the *Drosophila* ortholog of mammalian Nemo-like kinase (NLK), as a critical determinant of circadian clock speed [371,390]. Using an enhancer trap, Yu et al. (2011) mapped *nemo* expression to a subset of s-LN_v_s, l-LN_v_s, LN_d_s, and DN_1_s; however, it was absent in DN_2_ and DN_3_ neurons [390]. Knockdown of *nemo* in *tim*- or *Pdf*-expressing cells resulted in a shortening of behavioral period by ~2 h [371,390]. Chiu et al. (2011) demonstrated that NEMO phosphorylates Ser596 of PER [371]. This residue belongs in a phospho-cluster (Ser/Thr 583-596) that encompasses the *per^S^* mutation [366,370]. Using site-directed mutagenesis and biochemical approaches, Chiu et al. (2011) showed that abrogating phosphorylation of Ser/Thr residues within this cluster destabilized PER protein by accelerating the kinetics of DBT-mediated phosphorylation at other sites, including Ser47 [371]. Phosphorylation of Ser596 by NEMO primes PER for subsequent DBT-mediated phosphorylation within the phosphocluster (Ser585, Ser596) and the SLIMB-binding site (Ser47) [371]. Mutating Ser47 to alanine was sufficient to block PER(S596A)-induced destabilization of the protein [371]. Mutating the Ser/Thr residues within the phospho-cluster to alanine, either singly or in combination, resulted in ultra-short behavioral rhythms with periods of ~16 h [371]. Chiu et al. (2011) concluded that NEMO is the priming kinase for the *per^S^* phospho-cluster, which imposes a time delay on DBT-mediated phosphorylation at distal sites, the consequent interaction with SLIMB, and the daily downswing in PER levels [371]. On the other hand, data from Yu et al. (2011) suggested that NEMO may directly phosphorylate other components of the core clock machinery, including CLK [390]. 

### 5.5. PKA

A potential role of PKA in *Drosophila* circadian rhythms was first suggested by Levine et al. (1994), who observed that flies bearing mutations in the *dco* gene, which encodes the major catalytic subunit of PKA known as PKA-C1, were behaviorally arrhythmic [391]. The arrhythmia phenotype was observed by Majercak et al. (1997) in an independent *dco* mutant strain [392]. However, this study suggested that PKA may be functioning in clock output pathways rather than regulating the core clock itself, as the cycling of *per* at the mRNA and protein levels did not appear to be perturbed in their *dco* mutant flies [392]. Park et al. (2000) subsequently identified a fly strain deficient for a regulatory subunit of PKA II (PKA-RII) that also displayed arrhythmic locomotor activity [393]. Recently, Li et al. (2014) provided evidence to implicate PKA in the regulation of the molecular clock [288]. In *Pdf^01^* mutant flies, PER protein oscillations are severely dampened in the DN_1_s and LN_v_s after several days in DD [288]. The authors speculated that PDF stabilized PER via a cAMP/PKA-dependent mechanism [288]. In line with their hypothesis, treating isolated fly brains with the PKA activator, Sp-cAMPS, dramatically increased PER levels in the LN_v_s, a result that was mirrored in S2 cells following addition of Sp-cAMPS or forskolin, an activator of adenylyl cyclase [288]. PDF treatment also stabilized PER in isolated fly brains in a manner that was reversed by addition of the PKA inhibitor, PKI [288]. These results suggest that a PDF-cAMP-PKA pathway is important for promoting synchronization of clock neurons by stabilizing PER protein. 

### 5.6. p38 MAPK

In contrast with mammalian p38, there is clear evidence that p38 MAPK has a functional role in the *Drosophila* clock. The two p38 isoforms, p38a and p38b, are expressed broadly in the fly brain; within the clock network, their expression was detected in the small and large LN_v_s, DN_1a_s, and DN_3_s (p38b only) [394,395]. Within the DN_1a_ population, phosphorylation of p38 was rhythmic and clock-controlled, peaking in the subjective night [394]. Knocking down p38 in *tim*-expressing cells resulted in behavioral arrhythmicity in the majority of transgenic flies, whereas restricting the knockdown to PDF^+^ neurons prolonged the free-running period and delayed the onset of evening activity [394]. The period lengthening and delayed evening onset were recapitulated in flies that overexpressed a kinase-dead version of p38b in PDF^+^ or TIM^+^ cells [394]. Knockdown of p38a in *Pdf*- or *tim*-expressing cells phenocopied the p38b mutant flies, albeit the effects were milder [394]. Null mutants of either p38a or p38b had no effect on period length or rhythmicity, suggesting that the isoforms have redundant and compensatory functions [394]. Vrailas-Mortimer et al. (2014) reported a similar period lengthening in transgenic flies expressing a kinase-dead or a phospho-deficient mutant version of p38b, as well as (paradoxically) flies overexpressing the wild-type form of p38 [395]. In in vitro assays, p38b was shown to phosphorylate PER, likely at Ser661 (a priming site for hyperphosphorylation of PER) and Ser975 [394]. These results are consistent with reduced PER phosphorylation during the night in head extracts of flies expressing dominant-negative p38b in all clock cells [394]. The reduction in phosphorylated PER also predictably correlated with a delayed phase of nuclear PER accumulation in the small and large LN_v_s of these p38b mutant flies [394]. Overall, these two studies show that p38 has an integral role in regulating molecular clock oscillations by controlling PER phosphorylation and the timing of PER nuclear entry.

### 5.7. Ribosomal S6 kinase (RSK)

The *Drosophila* ortholog of RSK1, S6KII, has been shown in several studies to influence the pace of the fly clock through potentially divergent mechanisms. The expression of *S6KII* mRNA in the whole fly head is rhythmic, peaking in the late night to early morning [396]. A null mutation of S6KII did not alter light-induced phase delays or advances but shortened the behavioral period by ~1 h [396,397]. Constitutively overexpressing S6KII in *tim*- or *Pdf*-positive cells rescued the period phenotype of S6KII-null flies, suggesting that the expression, but not the oscillation, of S6KII is important for the circadian function of this kinase [396]. Transcript and protein levels of *per* were decreased and increased, respectively, in whole head extracts of S6KII-null flies, suggesting that there may be enhanced feedback repression in the absence of S6KII [396,397]. Akten et al. (2009) provided evidence to suggest that S6KII may be indirectly influencing the phosphorylation and stability of PER via CK2 [396]. S6KII-null flies carrying the *andante* mutation exhibited a long-period phenotype similar to the *andante* single mutants, while S6KII-null flies with the *Tik* mutation had a period that was also similar to, albeit slightly shorter than, the *Tik* single mutants [396]. These findings, along with the observation that S6KII physically associates with CK2, imply that S6KII is acting as an upstream inhibitor of CK2, affecting its ability to phosphorylate PER [396]. On the other hand, data from Beck et al. (2018) suggest an alternate mechanism involving SGG [397]. The C-terminal kinase domain (CTKD) of S6KII was shown to phosphorylate SGG at the inhibitory residue, Ser9 [397]. This is in line with previous observations demonstrating that overexpression of a kinase-dead version of the CTKD of S6KII did not rescue the short-period phenotype of S6KII-null flies [398]. As expected, S6KII-null flies exhibited enhanced SGG activity in whole head extracts and in the s-LN_v_s [397]. Importantly, the shortened period, enhanced PER accumulation, and reduced *per* transcript abundance of S6KII-null flies were rescued by reducing SGG expression through the introduction of a single copy of a loss-of-function *sgg* allele [397]. These findings suggest that S6KII is phosphorylating and inactivating SGG to slow the pace of the fly clock.

### 5.8. Ras/MAPK

Initial evidence that Ras/MAPK signaling may be implicated in the regulation of circadian rhythms in *Drosophila* came from the studies of neurofibromatosis-1 (*Nf1*) mutant flies [399]. Neurofibromin, the product of *Nf1*, is a GTPase-activating protein for Ras, promoting its inactivation by accelerating GTP hydrolysis. *Nf1*-deficient flies were behaviorally arrhythmic with no accompanying change in *per* or *tim* oscillations at the transcript or protein level, suggesting that *Nf1* is functioning within an output pathway [399]. As expected, levels of phospho-MAPK were elevated and the oscillatory amplitude of a CRE-luciferase reporter was significantly reduced in *Nf1* mutants [399]. Phospho-MAPK was detected in the dorsal brain in vicinity of PDF nerve terminals, and coincided with the time of maximal PDF release, suggesting that PDF activates Ras/MAPK signaling in PDF target cells [399]. A connection to the core clock machinery was first suggested by Weber et al. (2006), who showed that the transcriptional activity of CLK/CYC was enhanced by overexpressing a constitutively active form of Ras, or MEK1, in vitro [400]. In vitro assays revealed that CLK could be phosphorylated by ERK2 (and also CAMKII) [400]. Further evidence came from the study of S6KII-null flies, whose short-period phenotype could not be rescued by a mutant form of S6KII that was unable to bind ERK [398]. ERK binding appeared to be essential for autophosphorylation of S6KII at Ser515, a phosphorylation event that underlies the ability of S6KII to rescue the short-period phenotype of S6KII-null flies [398]. 

### 5.9. TOR

One study by Zheng and Sehgal (2010) implicated the TOR-ribosomal S6 kinase (S6K) signaling pathway in establishment of period in *Drosophila* pacemaker neurons [401]. Behavioral rhythms were lengthened by overexpression of any one of the following proteins in PDF neurons: TOR; its upstream activators, the protein kinase AKT or the GTPase Rheb; or its downstream effector, S6K [401]. Conversely, *Akt* hypomorphic mutants exhibited a shortened period [401]. However, co-expression of an active form of AKT and Rheb had an additive effect on period length, suggesting that AKT and Rheb/TOR/S6K (ie., TORC1) are signaling via independent pathways to influence period [401]. Overexpression of TOR, Rheb, or S6K induced the phosphorylation, and thus inactivation, of SGG [401]. This coincided with delayed nuclear accumulation of TIM in s-LN_v_s of mutant flies in which TOR signaling is enhanced in PDF neurons, presumably from reduced SGG-mediated phosphorylation of TIM [401]. The authors suggested that AKT and TOR signaling converged on SGG to regulate the timing of nuclear accumulation of TIM, thereby influencing circadian period. 

### 5.10. AMPK/SIK3

A recent study by Cho et al. (2019) implicated AMPK in the regulation of circadian rhythms in Drosophila [402]. The AMPK holoenzyme consists of a catalytic α subunit and two regulatory β and γ subunits, each encoded by a single gene in the fly genome. Knocking down each subunit in *tim*-expressing cells altered locomotor activity rhythms [402]. AMPKα and AMPKβ knockdown delayed the phase of evening anticipatory activity under LD and lengthened the period under DD [402]. AMPKγ knockdown advanced the evening peak under LD and resulted in arrhythmicity under DD [402]. The arrhythmic phenotype of AMPKγ mutant flies was attributed to a severe defect in the morphological integrity of the s-LN_v_s, which appeared to be missing the dorsally projecting neurites [402]. Adult-restricted silencing of AMPKγ reduced rhythmicity and shortened the period without affecting s-LN_v_ integrity, indicating that AMPKγ has a role in the adult clock in addition to a developmental function [402]. Compared to AMPKγ, AMPKα and AMPKβ knockdown had milder effects on the morphology of the s-LN_v_s [402]. In all AMPK mutants, PER abundance was reduced in the l-LN_v_s, LN_d_s, and DN_1_s [402]. In the case of AMPKβ, knocking down this subunit reduced the abundance of CLK in the s-LN_v_s and LN_d_s, suggesting that AMPK stabilizes CLK and promotes CLK-dependent transcription [402]. As expected, CLK overexpression in *Pdf*-expressing cells rescued the period lengthening induced by AMPKβ knockdown in these cells [402]. 

Similar to its mammalian counterpart, *Drosophila* SIK3 also has a functional role in clock neurons. Knocking down SIK3 in the LN_v_s lengthened the period of singly-housed male flies, whereas its silencing in the DN_1_s modestly shortened the period [403]. Interestingly, the male sex drive rhythm (MSDR), which is driven by a circuit of ~2000 neurons that include the LN_v_s, LN_d_s, and DN_1_s, was even more sensitive to the effects of SIK3 knockdown, consistently exhibiting a shortened period when SIK3 was inhibited in *fruitless*-expressing neurons or subsets of clock neurons [403]. Knocking down SIK3 in *Pdf*-expressing cells reduced the amplitude of PER oscillations in DN_1_ neurons, but not in the LN_v_s or LN_d_s: this effect may be mediated through disrupted communication between the LN_v_s and DN_1_s [403]. The mechanisms that couple SIK3 to the molecular clock remain unclear, but may involve SIK3-regulated nucleocytoplasmic trafficking of histone deacetylase 4 (HDAC4) [403]. 

### 5.11. GPRK2

No study to date has implicated the GRKs in the regulation of the central clock of *Drosophila*. However, *Drosophila Gprk2*, which closely resembles mammalian GRK4, was found to be expressed in a rhythmic manner in olfactory sensory neurons [404]. GPRK2 protein rhythms coincided with rhythms in electroantennogram (EAG) responses to ethyl acetate, and overexpression of GPRK2 promoted dendritic localization of odorant receptors [404]. These findings are consistent with a model whereby rhythmic GPRK2 expression enhances dendritic localization of odorant receptors, which in turn drives circadian rhythms in EAG response in olfactory neurons [404].

## 6. Concluding Remarks

Although the mammalian SCN and the *Drosophila* clock network differ markedly in their structural organization, the similarities between the two systems become more apparent when molecular comparisons are made. One of the most obvious similarities is the role that protein kinases play in phosphorylating core clock proteins and controlling the kinetics of their degradation; in doing so, protein kinases in both systems have proven to play a pivotal role in determining circadian period. Several orthologous kinases (e.g., CK1δ/ε and DBT, or GSK3 and SGG) are remarkably similar in their effects on period, raising the possibility that other sets of kinase orthologs may prove, upon closer scrutiny, to have parallel functions in flies and mammals. The commonalities in intercellular communication are also noteworthy, with receptor orthologs playing broadly similar functions in both systems. The most obvious example is the comparison between PDFR and VPAC2. There is great potential for knowledge translation between the mammalian and *Drosophila* circadian systems, such that understanding the signaling mechanisms in one may reveal insights into the other.
